# Cellular and immune adaptations at the maternal-fetal interface in bats

**DOI:** 10.1016/j.celrep.2025.116645

**Published:** 2025-12-15

**Authors:** Allyson Caldwell, Liheng Yang, Rebecca L. Casazza, Rizban E. Worota, Cole McCutcheon, Patrick S. Creisher, Erika Zhan, Clara Reasoner, Ashley Higgins, Tony Schountz, Carolyn B. Coyne

**Affiliations:** 1 Duke University School of Medicine, Department of Integrative Immunobiology, Durham, NC 27710, USA; 2 Department of Microbiology, Immunology and Pathology, College of Veterinary Medicine, Colorado State University, Fort Collins, CO 80523, USA; 3 Duke Human Vaccine Institute, Durham, NC 27710, USA; 4 These authors contributed equally; 5 Lead contact

## Abstract

Bats experience extreme physiological conditions rarely encountered by other mammals, including prolonged gestation relative to other small species, high metabolic demands, temperature fluctuations during flight, and continual microbial exposure. These traits make them a powerful model for understanding placental adaptation during pregnancy. Here, we define the cellular and molecular architecture of the Jamaican fruit bat (*Artibeus jamaicensis*) placenta using single-nucleus RNA sequencing and tissue-derived organoid models. This analysis reveals diverse trophoblast, stromal, and immune populations with bat-specific transcriptional programs, including fibroblasts with hybrid adventitial and neuronal signatures and macrophages expressing pregnancy-associated molecules typically restricted to trophoblasts. Comparative analyses with human and mouse placentas uncover both conserved and lineage-specific features. Functional assays demonstrate that bat trophoblast organoids maintain high basal antiviral gene expression but limited inducibility following viral stimulation, revealing a unique strategy of immune vigilance without inflammation at the maternal-fetal barrier that may underpin reproductive success under physiological extremes.

## In brief

Caldwell, Yang, Casazza, et al. map the cellular landscape of the Jamaican fruit bat placenta using single-nucleus RNA sequencing, immunohistochemistry, and confocal imaging. They identify diverse trophoblast, stromal, and immune populations and develop trophoblast and decidua organoids that enable *in vitro* modeling of bat placental biology.

## Graphical Abstract

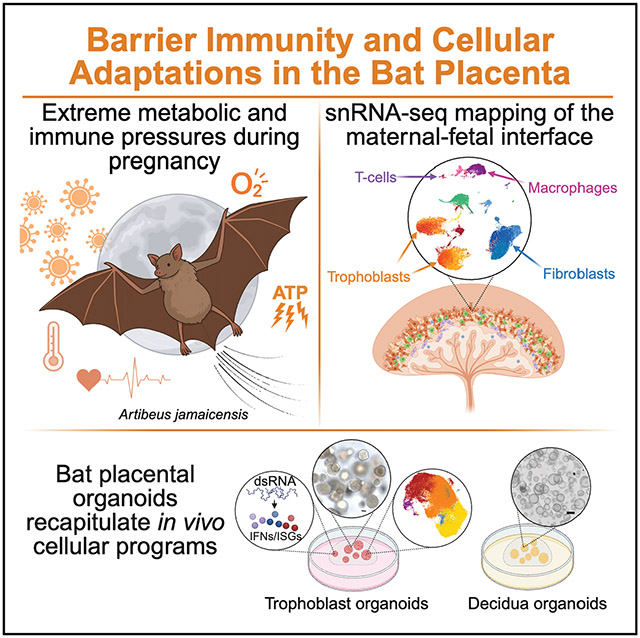

## INTRODUCTION

Mammalian pregnancy requires extensive physiological, cellular, and immune adaptations to support fetal development while preserving maternal health. These adaptations vary across species and are shaped by gestation length, litter size, maternal immune architecture, and ecological pressures. Central to reproductive success is the placenta, a transient organ mediating nutrient and gas exchange, hormone production, and immune tolerance at the maternal-fetal interface. Although all placentas serve these core functions, they display remarkable diversity, reflecting millions of years of evolutionary innovation across mammalian lineages.^1,2^

Bats provide a compelling system for investigating how the placenta adapts to physiological extremes. As the second-largest mammalian order with more than 1,400 species, bats exhibit extraordinary variation in placental morphology and reproductive strategy.^3^ Many sustain prolonged gestation relative to body size, with some employing delayed implantation or developmental suspension to synchronize birth with environmental cues. Unlike most small mammals that produce shortgestation litters, many bats give birth to a single offspring after extended intrauterine development, suggesting that their placentas must support sustained fetal growth under distinct mechanical and metabolic constraints.

Bats also experience physiological and immunological challenges that place unique demands on placental function. Sustained flight imposes high metabolic rates and oxidative stress, whereas torpor introduces fluctuating thermal and hemodynamic states.^4^ Dense roosting and high population mobility elevate pathogen exposure in bats, which serve as natural reservoirs for a wide range of zoonotic viruses, including coronaviruses, filoviruses, and henipaviruses, yet show no signs of disease.^5-8^ Studies reveal dampened activation of interferon, nuclear factor κB (NF-κB), and inflammasome pathways despite constitutive antiviral gene expression.^9-11^ This tolerance to persistent viral presence raises key questions about how systemic immune adaptations are integrated into placental biology, where immune equilibrium is critical for fetal survival.

Prior work on bat placentation has largely focused on morphology. Classical studies described vascular and membrane organization in *Desmodus rotundus* and other species,^12-15^ showing that most microbats possess hemomonochorial placentas with a single syncytial trophoblast (TB) layer contacting maternal blood. Some species exhibit dynamic changes in placental structure throughout gestation, transitioning from endotheliochorial to hemodichorial forms.^16^ However, cellular and molecular mechanisms that govern bat placental development, TB lineage specification, immune recruitment, and nutrient exchange remain unknown.

TBs are the defining cell lineage of the placenta, but their differentiation trajectories and functions vary widely across mammals. In hemochorial placentas, such as those in humans, cytotrophoblast (CTB) progenitors give rise to the multinucleated syncytiotrophoblast (STB) that mediates nutrient, gas, and waste exchange, as well as to invasive extravillous TBs (ETVs) that remodel maternal vasculature. Additional cell types at the maternal-fetal interface, including stromal fibroblasts (FBs), endothelial cells (Endos), glandular epithelium (Gland-EpC), and diverse immune populations, act together to support implantation, fetal tolerance, and tissue remodeling. Although these lineages have been well defined in human and murine placentas, their composition, developmental dynamics, and transcriptional programs remain uncharacterized in bats. As a result, the cellular basis for placental specialization in this lineage, including how bats sustain gestation, tolerate fetal antigens, and resist infection at the maternal-fetal interface, remains largely unknown.

To address these gaps, we combined single-nucleus RNA sequencing (snRNA-seq) and tissue-derived organoid models to map the cellular and molecular architecture of the Jamaican fruit bat (Jfb; *Artibeus jamaicensis*) placenta. snRNA-seq defined the full spectrum of TB, stromal, endothelial, and immune populations, while matched organoid models captured key TB lineages and allowed *in vitro* reconstruction of differentiation programs. This integrative approach revealed bat-specific cellular adaptations. Cross-species comparisons with human and mouse placentas uncovered both conserved and lineage-specific features, providing insight into the evolution of placental biology, TB specialization, and maternal-fetal immune regulation.

## RESULTS

### Isolation and characterization of Jfb placenta

Placentas were collected from five visibly pregnant adult female Jfbs (family Phyllostomidae) housed in a controlled colony and euthanized under approved animal protocols. The Jfb was selected for its accessibility and reliable breeding in captivity, enabling consistent collection of mid- to late-gestation samples. The gestational stage was estimated using established morphological criteria for phyllostomid bats,^17^ including fetal crownrump length (CRL), wing digit separation, membrane translucency, and the ratio of placental disc to fetal size. Features such as eye reopening, pigmentation of skin and claws, elongated digits, and a ribbed nose leaf indicated stage 21–24 development, with one placenta (Plac1, stage 24) in the fetal period based on CRL, skin wrinkling, and pigmentation (Figures S1A and S1B). Fetal sex was determined by external genital morphology, based on the presence or absence of a midline genital tubercle caudal to the umbilicus.^18^ Three fetuses were male (Plac2, Plac4, and Plac5) and two were female (Plac1 and Plac3) (Figure S1B).

**Figure 1.**
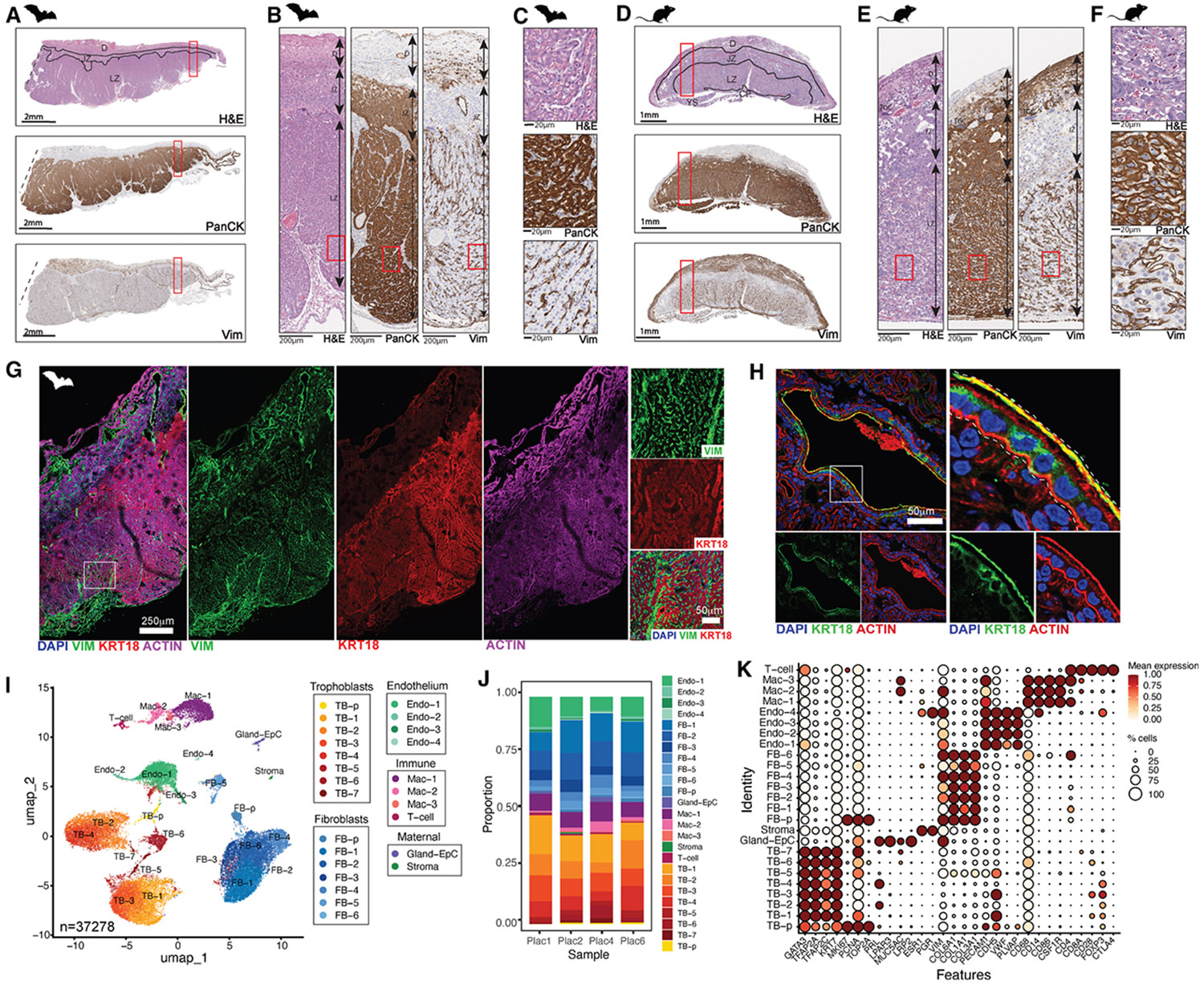
Cellular landscape of the Jamaican fruit bat placenta revealed by histology, immunohistochemistry, and snRNA-seq (A–F) Histological and immunohistochemical staining of Jamaican fruit bat (Jfb) (A–C) and mouse (D–F) placentas. Low-magnification (A and D) and high-magnification (C and F) images show hematoxylin and eosin (H&E) (top), pan-cytokeratin (Pan-CK) (middle), and vimentin (Vim) (bottom) staining. Placental regions—decidua (D), labyrinth zone (LZ), junctional zone (JZ), and yolk sac (YS)—are labeled and outlined by black lines in (A) and (D). (B and E) Zoomed crosssections of corresponding regions. The scale bars are shown at the bottom. (G) Stitched tile-scan confocal micrograph of Jfb placenta immunostained for VIM (green), KRT18 (red), and actin (purple); nuclei are DAPI (blue). Left: mosaic overview. Right: magnified boxed region. (H) Confocal micrograph of Jfb single syncytiotrophoblast (STB) layer immunostained for KRT18 (green) and actin (red). The scale bar is shown at the bottom. On the right are zoomed images from the white box shown on the left. White hatched lines mark a single STB layer. (I) UMAP of snRNA-seq data from four Jfb placentas (*n* = 37,278 nuclei). (J) Sample contribution to the 25 clusters. (K) Dot plot of canonical gene expression used to assign cluster identities. The scale is shown on the right. All IHC panels represent data from five biological replicates; IF analyses were performed on three independent placentas, with representative images shown.

To characterize placental morphology, bat tissue was processed for histology alongside late-gestation (~embryonic day [E]17.5) mouse placentas. Hematoxylin and eosin staining revealed a large, highly vascularized labyrinth occupying ~two-thirds of the bat placenta, morphologically analogous to the murine labyrinth zone (Figures 1A-1F). Immunohistochemistry (IHC) using pan-cytokeratin (Pan-CK) or cytokeratin 18 (KRT18) identified two TB regions: a vascularized labyrinth and a narrow band of distinct TBs between the labyrinth and decidua. We refer to this intermediate region as the junctional zone, consistent with murine nomenclature (Figures 1A-1F, middle, and S1E). Although similarly positioned, bat junctional TBs were structurally distinct from their murine counterparts, suggesting unique biology. Within this layer, cells adjacent to the labyrinth and decidua differed in morphology, indicating multiple TB subtypes (Figures 1B and 1E). Periodic acid-Schiff (PAS) staining detected glycogen in junctional TBs and decidual cells of the bat placenta, with comparable but fainter staining in mouse glycogen TBs and decidua (Figures S1C and S1D). While this suggests conserved glycogen storage, the extent of PAS staining in bats may vary with gestational stage. Endothelial and fibroblast (FB) populations were visualized with anti-vimentin, which labeled fetal microvasculature in the bat labyrinth similarly to that of mice (Figures 1A, 1C, 1D, and 1F and Figures 1B and 1E, respectively). Lack of vimentin staining further delineated the junctional zone, and combined cytokeratin/vimentin staining highlighted dual blood supplies in both species, separated by narrow layers of fetal endothelium and STB (Figures 1C and 1F). A high-resolution stitched confocal scan of KRT18/vimentin co-staining delineated maternal and fetal vascular compartments (Figure 1G). Higher magnification revealed a continuous STB layer separating maternal blood from the fetal endothelium, confirming a hemomonochorial placenta (Figure 1H). This architecture is consistent with prior reports in other phyllostomid bats^15^ and supports an invasive placental organization marked by direct maternal blood contact with the STB layer.

### Single-nucleus transcriptomic profiling of the Jfb placenta

Building on these histological findings, we next sought to define the cellular and transcriptional landscape of the Jfb placenta using snRNA-seq. This approach was chosen based on previous findings that snRNA-seq is necessary to capture the STB in human placental tissue,^19,20^ which is often underrepresented in single-cell datasets due to its large, multinucleated structure and fragility during dissociation. Nuclei were isolated from four placentas, including one near full term (Plac1) and three from mid- to late-gestation stages (Plac2, Plac4, and Plac6). Following quality control and filtering, we obtained a total of 37,278 high-quality nuclei for downstream analyses. This analysis resolved 25 transcriptionally distinct clusters across all samples, present in samples at near-equivalent ratios and expressing distinct cluster-associated genes (Figures 1I, 1J, and S2C; Table S1). We determined the optimal clustering resolution using silhouette score analysis^21^ as a quantitative measure of cluster separation. This analysis yielded a positive average silhouette width (ASW ≈ 0.287), indicating that the cells were, on average, closer to their assigned cluster than to any other cluster, thereby supporting the chosen resolution (Figures S2A and S2B). Moreover, the observed silhouette distribution was significantly shifted upward relative to a random-label baseline, confirming that the resulting clusters capture non-random, biologically meaningful structures (Figure S2A). There were no significant differences between the late-gestation female Plac1 sample and those samples from earlier-gestation males (Figures 1J and S2C). To assign celltype identities to the 25 transcriptional clusters, we used a curated panel of canonical marker genes based on well-characterized human placental and immune cell populations^19,20,22,23^ (Figure 1K). Although the data were derived from Jfb tissue, these conserved markers provided a robust framework for interpreting bat placental cell types. TB clusters were identified by expression of lineage-defining genes, such as *GATA3* and *KRT7*. Stromal and FB populations were distinguished by expression of *ESR1*, *PGR*, and extracellular matrix (ECM) genes. Actively cycling cells were marked by proliferation-associated genes such as *MKI67* and *PCNA*. Endos expressed *PECAM1* and *PLVAP*, while macrophage (Mac) populations were identified by expression of *CD68* and *CSF1R*. T cell populations were annotated based on expression of *CD4* and *FOXP3*. A subset of clusters was also characterized by the expression of decidual gland-associated markers, including *PRL* and *CDH2*, consistent with secretory epithelial cell identity. The presence of decidual glands was supported by H&E staining, which revealed glandular structures in placentas with intact uterine sections (Figure S1F).

Using this panel of canonical markers, we assigned identities to transcriptional clusters corresponding to all major placental and maternal cell types in Jfb placentas. To further validate these annotations, we examined the top five uniquely enriched genes per cluster, which confirmed distinct transcriptional signatures consistent with discrete lineages (Figure S2D). We also assessed proliferative activity by calculating S-phase and G2/M-phase scores based on curated gene sets. Two clusters exhibited elevated scores for both phases, indicating high proliferative activity (Figure S2E). These clusters were subsequently annotated as TB-p and FB-p, representing proliferative progenitors of the TB and FB lineages, respectively. Based on these analyses, we defined eight TB clusters, comprising TB-p and seven additional subclusters (TB-1–TB-7) spanning a range of progenitor, transitional, and specialized states. The FB lineage consisted of seven clusters, including FB-p and FB-1–FB-6, which captured transcriptional heterogeneity among stromal and mesenchymal cells. Four endothelial clusters (Endo-1–Endo4) were defined by vascular markers, and three Mac clusters (Mac-1–Mac3) represented immune cell diversity within the placental microenvironment. A distinct T cell cluster and two maternal-derived clusters, Gland-EpC and stroma, were also identified. These lineage annotations are visualized in the uniform manifold approximation and projection (UMAP) (Figure 1I) and were distributed evenly across all samples (Figures 1J and S2C). Together, these data define a comprehensive single-nucleus atlas of the hemomonochorial Jfb placenta, linking placental architecture to molecularly distinct cell states.

### Identification of bat TB markers and pregnancy-specific glycoproteins

**Figure 2.**
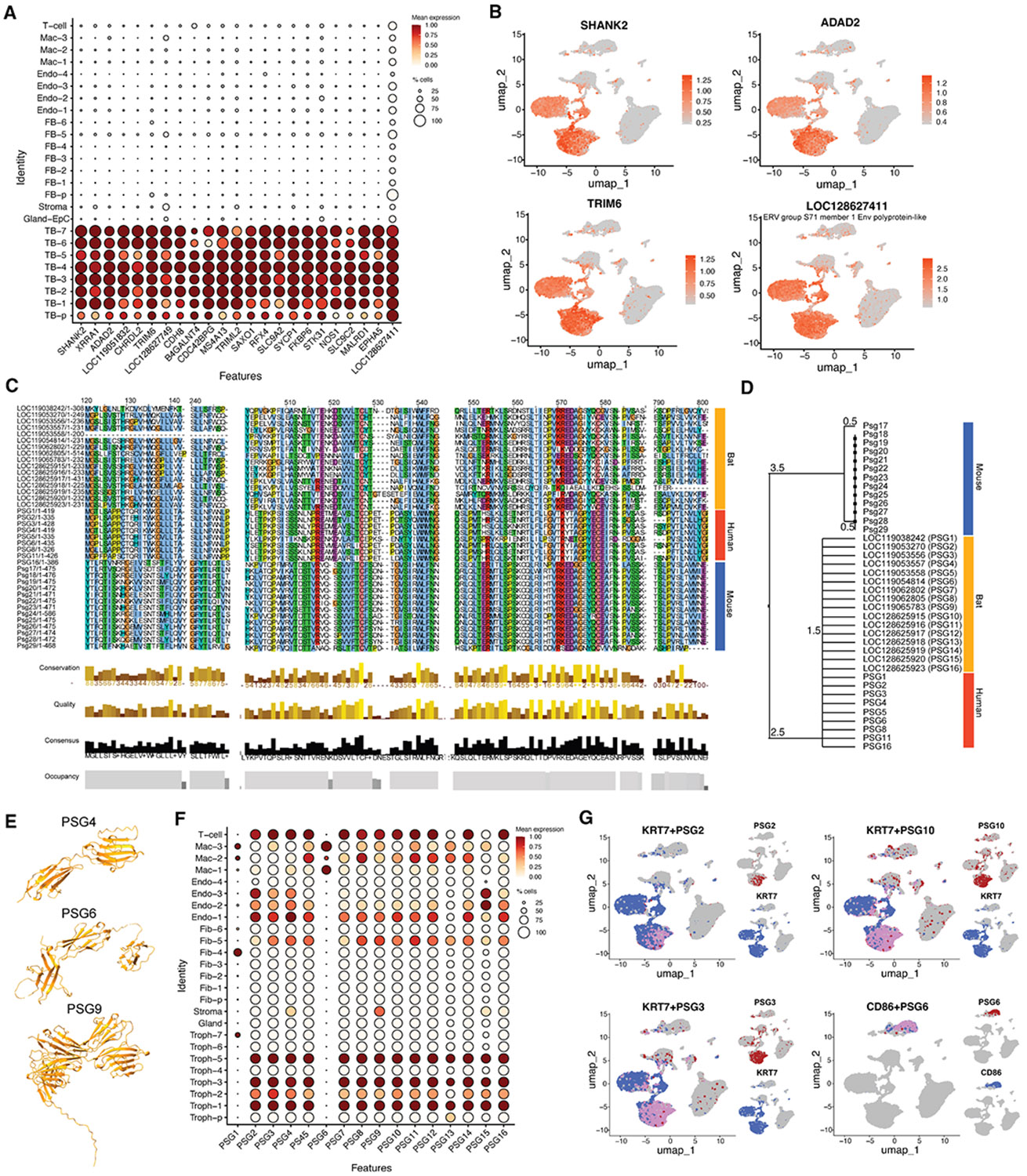
Identification of bat trophoblast markers and pregnancy-specific glycoproteins (A) Dot plot of genes enriched in Jfb placental trophoblasts versus non-trophoblasts determined by differential expression analysis. The key is shown on the right. (B) FeaturePlots of select genes identified in (A). The scale is shown on the right. (C) Conserved regions across pregnancy-specific glycoprotein (PSG) alignments of candidate bat PSGs (yellow-orange bar) and select human PSGs (red bar) or mouse Psgs (blue bar). (D) Phylogenetic tree based on ClustalW multiple sequence alignment of candidate bat PSGs with human and mouse PSG families. (E) Domain architecture of representative bat PSGs, predicted by AlphaFold3. (F) Dot plot of bat PSG expression across placental cell types. (G) FeaturePlots showing co-expression of a bat PSGs (in red) with KRT7 (blue) or expression of PSG6 with CD86 (blue, bottom right).

To define molecular signatures of bat TBs at single-nucleus resolution, we classified all nuclei as TB or non-TB based on marker expression (Figure S2F) and performed differential gene expression analysis using the MAST framework. This revealed ~3,700 TB-enriched genes (coding and non-coding; log_2_ fold change [log_2_FC] > 2, adjusted *p* [*padj*] < 0.05; Table S2). Because the *A. jamaicensis* genome includes redundant LOC annotations, the true number of unique genes is likely lower. The gene set encompassed known placental regulators and previously uncharacterized loci (Figures 2A and 2B). For example, *SHANK2*, a scaffolding protein with potential structural or signaling roles in TBs, and *CHRDL2*, a BMP antagonist possibly regulating differentiation. Other enriched transcripts included solute carriers (*SLC9A2* and *SLC9C2*), transcription factors (*RFX4*), and endogenous retroviral elements, such as *LOC128627411*, annotated as a group S71 envelope polyprotein-like gene. Together, these findings establish a broad repertoire of bat-specific TB genes and provide a foundation for understanding species-specific placental adaptations.

Pregnancy-specific glycoproteins (PSGs) represent one of the most prominent and lineage-restricted TB gene families in mammals. In humans, PSGs are secreted by the STB and modulate maternal immune responses while supporting fetal development,^24-29^ but they remain largely uncharacterized in bats. To explore this possibility, we used methods similar to those used previously to identify PSGs in other bat species.^30^ We searched the *A. jamaicensis* genome for annotated and unannotated carcinoembryonic antigen-related cell adhesion molecules (CEACAM)-like genes, identifying 31 PSG-like candidates (Table S3). To distinguish secreted PSGs from membranebound CEACAMs, we used DeepTMHMM to predict transmembrane domains,^31^ identifying 24 soluble candidates consistent with PSG identity. To assess orthology, we aligned these 24 bat PSG candidates with human (PSG1–PSG8, PSG11, and PSG16) and mouse (Psg17–Psg29) sequences using ClustalW. Sixteen genes showed strong sequence consensus and were designated as high-confidence bat PSGs (Table S3). Jalview^32^ visualizations confirmed conserved motifs, and MEGA11 phylogenetic analysis^33^ showed that bat PSGs cluster more closely with human than with mouse PSGs, suggesting greater conservation with the primate lineage (Figures 2C and 2D). AlphaFold3 predictions^34^ revealed preservation of immunoglobulin (Ig)-like domain architecture, an N-terminal IgV domain followed by one or more IgC domains, characteristic of bona fide PSGs (Videos S1 and S2; Figure 2E). Based on these analyses, we designated these 16 genes as bat PSG1–PSG16, representing a previously unrecognized expansion of the PSG family in bats. Expression mapping across the full snRNA-seq dataset confirmed that most bat PSGs are robustly expressed in TB clusters and colocalize with KRT7 (Figures 2F and 2G). Intriguingly, PSG6 displayed a distinct expression pattern restricted to a Mac population (Figure 2G), suggesting that certain bat PSGs may have evolved noncanonical, immunoregulatory roles beyond the TB lineage, contributing to bat maternal-fetal immune balance.

### TB diversity and specialization in the Jfb placenta

**Figure 3.**
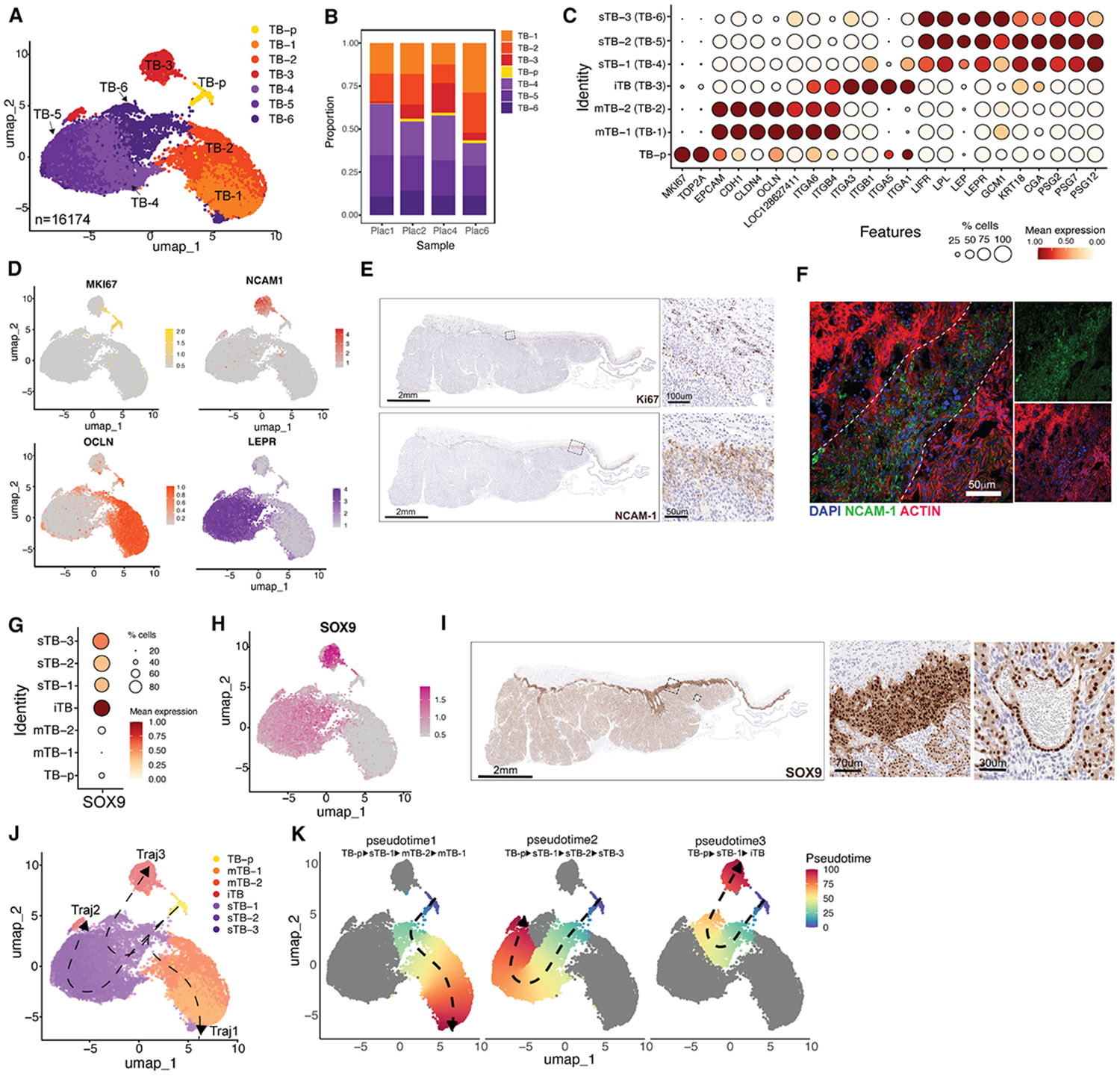
Transcriptional heterogeneity and differentiation trajectories of trophoblast populations in the Jamaican fruit bat placenta (A) UMAP of subsetted trophoblast populations from snRNA-seq data, resolving seven clusters. (B) Sample contribution to each trophoblast cluster. (C) Dot plot of canonical human trophoblast subtype markers and select bat PSGs across bat trophoblast clusters, resolving cluster identities in (A). (D) FeaturePlots of proliferative (*MKI67*), epithelial (*OCLN*), invasive (*NCAM1*), and syncytial (*LEPR)* states. (E) Immunohistochemistry for Ki67 and NCAM1 on Jfb placental sections. (F) Confocal micrograph of NCAM-1 (in green) and actin (in red), confirming the localization of NCAM-1 to invasive trophoblasts in the junctional zone, labeled with hatched white lines. Scale bar, 50μm. (G and H) Dot plot of SOX9 expression (G) and FeaturePlot (H). (I) Immunohistochemistry for SOX9 in bat placenta. Images on the right are zoomed from the black boxes on the left. The scale is shown at the bottom. (J) Slingshot trajectory analysis of trophoblast populations, revealing three major lineages. (K) UMAP of trophoblast differentiation trajectories, colored by pseudotime (rainbow scale) and with arrows indicating directionality; red denotes terminal differentiation states. All IHC panels represent data from two biological replicates; IF analyses were performed on three independent placentas, with representative images shown.

To dissect TB diversity in the Jfb placenta, we reanalyzed TB-enriched clusters to resolve transcriptional states and infer lineage relationships. Using silhouette score analysis,^21^ we identified seven transcriptionally distinct clusters (ASW ≈ 0.159), representing robust biological structures (Figures S3A-S3C). On the UMAP, TB1–TB2 formed one axis and TB4–TB6 another, with the proliferative TB-p cluster preserved from the global analysis (Figure 3A). The apparent spatial separation of these clusters reflects transcriptomic similarity rather than physical or temporal positioning, as UMAP coordinates do not imply spatial relationships. Cluster frequencies were consistent across placentas (Figures 3B and S3D), and S- and G2M-phase scoring confirmed that proliferative activity was largely restricted to TB-p (Figure S3E).

Each cluster exhibited distinct transcriptional signatures (Figures S3F and S3G): TB1–TB2 were enriched for signaling and WNT-related genes (e.g., *CHST4* and *WNT3*), TB3 expressed neuroendocrine and immune-modulatory genes (e.g., *PTPRN* and *GDNF*), and TB4–TB6 expressed metabolic and hormone-responsive genes (e.g., *CCKAR* and *PLA2G4D*). Gene Ontology (GO) enrichment analysis supported these functions, highlighting transcriptional regulation and hormone signaling pathways (Figure S3H).

To relate these clusters to known human TB types, we examined canonical CTB, EVT, and STB markers. TB1–TB2 expressed epithelial and progenitor markers (e.g., *EPCAM* and *CDH1*), consistent with mononuclear TBs (mTBs). TB3 showed strong expression of invasive and immunomodulatory genes (e.g., *ITGA1*, *ITGA5*, *NCAM1*, and *MMP14*), defining an invasive TB (iTB) state. TB4–TB6 expressed syncytial and hormone-related genes (e.g., *CGA* and *PSG*s), corresponding to syncytial TBs (sTBs) (Figures 3C and 3D). Based on these signatures, clusters were annotated as TB1–TB2 = mTB, TB3 = iTB, and TB4–TB6 = sTB. IHC and immunofluorescence (IF) validated these identities: Ki67 marked proliferative TB-p nuclei, while NCAM-1 localized to iTBs at the junctional zone (Figures 3E and 3F). Cross-cluster marker enrichment revealed SOX9 as a transcription factor enriched in iTBs and sTBs, with minimal expression in TB-p or mTBs (Figure 3G and 3H). IHC confirmed nuclear SOX9 in iTBs and sTBs (Figure 3I), identifying it as a candidate regulator of TB differentiation in the Jfb placenta.

To infer lineage relationships, we applied Slingshot trajectory analysis using TB-p as the root. Three developmental trajectories emerged: (1) TB-p → sTB-1 → mTB-2 → mTB-1, consistent with progenitor-to-CTB maturation; (2) TB-p → sTB-1 → sTB-2 → sTB-3, reflecting syncytial differentiation; and (3) TB-p → sTB-1 → iTB, indicating a shared intermediate with invasive lineages (Figures 3J and 3K). Notably, sTB-1 expressed proliferation markers, supporting its identification as an intermediate, proliferative state at the convergence of these trajectories (Figure S3G). fitGAM modeling revealed trajectory-specific expression dynamics, including *LEPR* induction along the syncytial branch and *NCAM1* upregulation along the invasive trajectory (Figure S3I). Collectively, these analyses define a continuum of TB differentiation in the Jfb placenta, with sTB-1 representing a transitional state giving rise to mononuclear, invasive, and syncytial lineages.

### Mapping immune cell populations in the Jfb placenta

**Figure 4.**
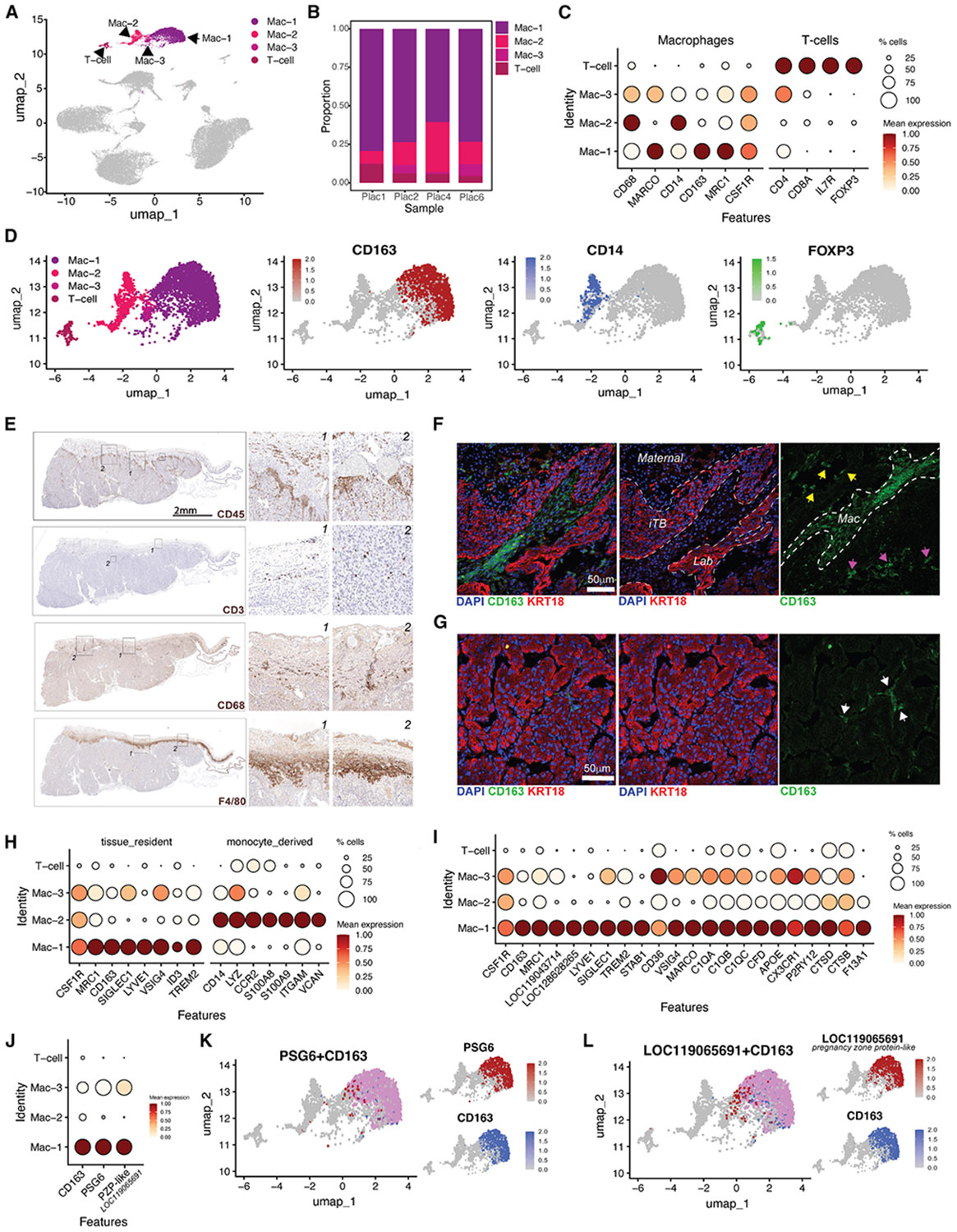
Immune cell diversity and functional specialization at the maternal-fetal interface of the Jamaican fruit bat placenta (A) UMAP of immune cell populations identified in Jamaican fruit bat placenta snRNA-seq data, resolving three macrophage clusters and one T cell cluster. (B) Proportional contribution of each sample to immune clusters. (C) Dot plot of canonical macrophage and T cell markers. (D) FeaturePlots of immune markers *CD163* (red), *CD14* (blue), and *FOXP3* (green) shown on a UMAP subsetted to dimensions capturing only immune cell populations (left), improving resolution of cluster-specific expression patterns. (E) Immunohistochemistry for CD45 (pan-leukocyte marker), CD3 (T cell marker), CD68, and F4/80 (macrophage markers). Scale bar, 2 mm. (F and G) Confocal micrographs of Jfb placental tissue stained for CD163 (green) and KRT18 (red). (F) CD163^+^ cells localize to the junctional zone between invasive trophoblasts (iTBs) and labyrinth (Lab); the boundary is indicated by white hatched lines (middle). On the right, CD163^+^ macrophages (Mac) are outlined with white dashed lines. Yellow arrows mark positive cells in the maternal compartment; pink arrows mark positive cells within Lab. Scale bar, 50μm. (G) CD163^+^ macrophages within Lab are marked by white arrows. Scale bars are shown at the bottom right of the images on the left. (H) Expression of tissue-resident versus monocyte-derived macrophage markers across macrophage clusters by dot plot. (I) Expression of Hofbauer cell-associated genes. (J) Dot plot showing differential enrichment of *LOC119065691* (a pregnancy zone protein [PZP]-like homolog) and *PSG6* in Mac-1 relative to other macrophage populations or T cells. (K and L) Co-expression of (I) *PSG6* (red) or (J) *LOC119065691/PZP-like* (red) and *CD163* (blue) within Mac-1 as assessed by FeaturePlot. All IHC panels represent data from three biological replicates; IF analyses were performed on three independent placentas, with representative images shown.

snRNA-seq identified three transcriptionally distinct Mac populations and one T cell population in the Jfb placenta (Figures 4A, 4B, and S4A; Table S5). All populations expressed canonical lineage markers supporting their identity and function within the placental environment (Figure 4C). Macs expressed *CD68* and *CSF1R*, while T cells expressed *CD4* and *FOXP3*. Marker expression revealed heterogeneity among Macs (e.g., *CD14* enrichment in one cluster and *CD163* in another), suggesting functional specialization (Figures 4C, 4D, and S4B). The T cell cluster exhibited a mixed transcriptional profile, co-expressing naive (e.g., *SELL* and *CCR7*), effector (e.g., *IFNG* and *PRF1*), and regulatory (e.g., *FOXP3* and *IL2RA*) markers, confirming a heterogeneous T cell identity (Figure S4B). To validate and localize immune cells *in situ*, we performed IHC for CD45 (panleukocyte), CD3e (T cells), CD68, and F4/80 (tissue-resident Macs). CD45^+^ cells were enriched in the junctional zone, particularly around the microvasculature, while CD3e^+^ T cells were rare but present in both the junctional zone and labyrinth (Figure 4E). CD68^+^ Macs were abundant in the junctional zone, and F4/80 showed strong, regionally restricted staining consistent with tissue-resident Macs. IF and confocal imaging with CD163 confirmed Mac localization, with CD163^+^ cells concentrated in the junctional zone and, to a lesser extent, in the labyrinth (Figures 4F and 4G), indicating spatial compartmentalization of resident Macs at the maternal-fetal interface.

To distinguish tissue-resident from monocyte-derived Macs, we analyzed curated marker panels. Mac-1 strongly expressed tissue-resident genes (e.g., *CSF1R* and *LYVE1*), Mac-3 expressed these at lower levels, and Mac-2 was enriched for monocyte-derived markers (e.g., *CD14* and *LYZ*) (Figure 4H). Given its high expression of tissue-resident markers, we next assessed whether Mac-1 resembled Hofbauer cells, the fetal Macs of the human placenta. Mac-1 expressed a Hofbauer-like gene signature,^22^ including *CSF1R* and *APOE* (Figure 4I), while Mac-2 and Mac-3 lacked this program, consistent with more inflammatory or recruited Mac states.

Marker enrichment analysis identified *LOC119065691*, encoding a pregnancy zone protein (*PZP*)-like homolog, as the top gene upregulated in Mac-1 (log_2_FC = 5.86; *padj* = 1.84 × 10^−260^) (Figure 4J; Table S5). *PZP* expression was highly restricted to Mac-1 and colocalized with CD163, a hallmark of tissue-resident, anti-inflammatory Macs (Figures 4J and 4K). Notably, *PSG6*, one of the 16 bat PSGs, was also selectively expressed in this cluster (Figures 4K and 4L). Together, these findings identify Mac-1 as a Hofbauer-like, fetal-derived Mac population distinguished by high *PZP* and *PSG6* expression, suggesting the evolution of a specialized, immunoregulatory Mac program within the bat placenta.

### Cross-species analysis of bat, human, and mouse placental cell types

**Figure 5.**
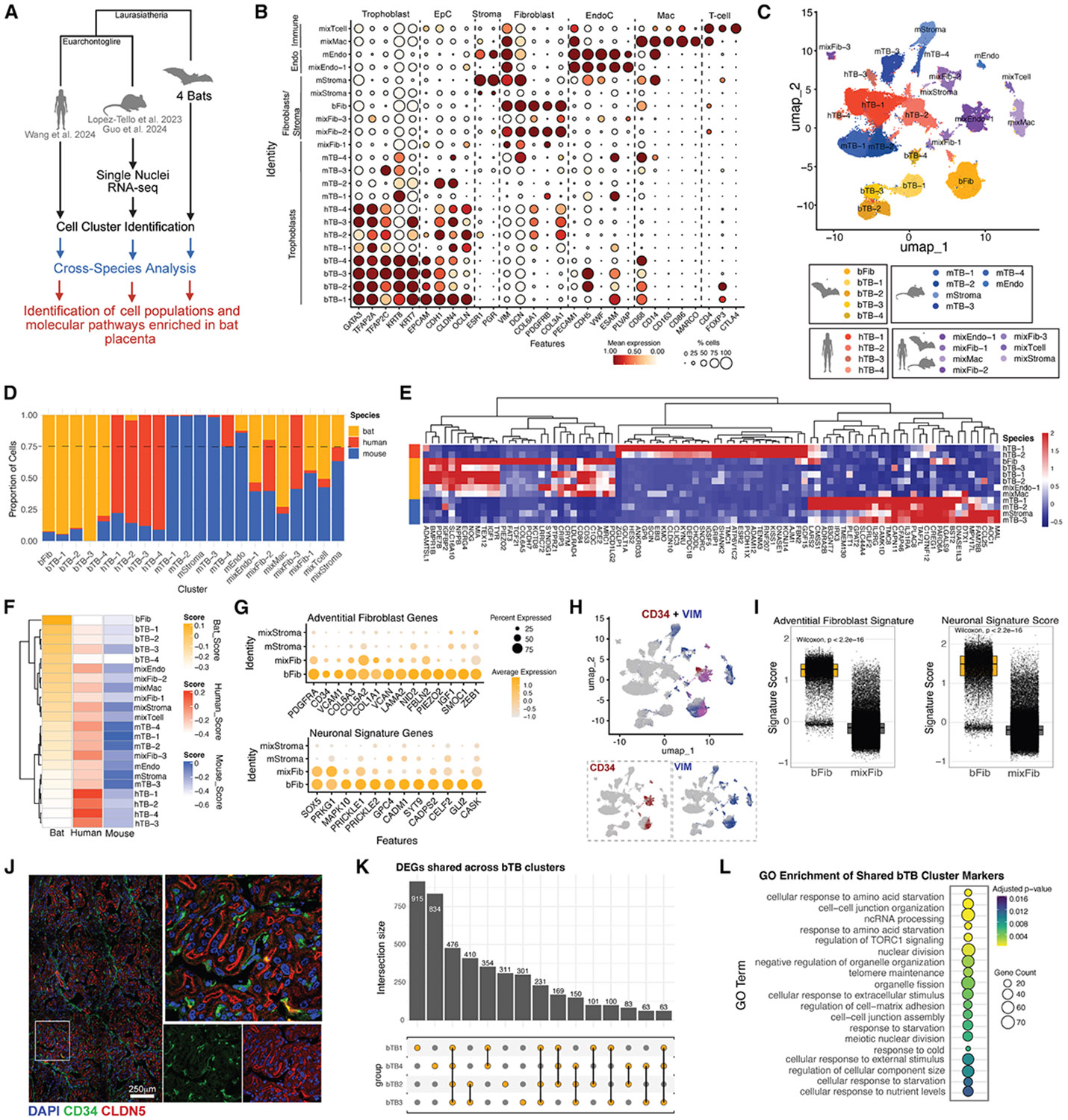
Cross-species single-nucleus RNA-seq integration, comparing placental datasets from Jamaican fruit bat, human, and mouse (A) Evolutionary relationships are shown on the left (mya, million years ago). (B) Dot plot of canonical genes across integrated clusters. Dot size reflects the percentage of expressing cells, and color indicates the scaled expression (the key is shown at the bottom). (C) UMAP projection of integrated datasets shows species-specific and mixed-species clusters. Bat-derived clusters (yellow-orange, “b”), human clusters (red, “h”), mouse clusters (blue, “m”), and mixed-species clusters (purple, “mix”) are shown. (D) Barplot showing species contribution to each cluster. (E) Heatmap displaying contribution of species-restricted gene signatures; the key is shown on the right. (F) Heatmaps show scaled expression (*Z* score) of bat-, human-, and mouse-specific gene signature scores across all annotated clusters. Signature scores were calculated per cell using the top 1,000 differentially expressed genes per species (MAST, false discovery rate [FDR] < 0.05, log_2_FC > 0.25) and then averaged by cluster. Each column represents a species-specific gene signature, and each row indicates a defined placental cluster. Row names denote cluster identity, and color intensity reflects relative enrichment within each cluster. The cluster order is maintained across species. (G) Dot plots of select genes from adventitial (top) and neuronal (bottom) gene sets comparing expression in bat-specific fibroblasts (bFibs), mixed-species fibroblasts (mixFibs), and stromal populations, with the key shown on the right. (H) FeaturePlots of CD34 (red) and VIM (blue) on the UMAP embedding. Co-expression appears purple (top), and individual gene expression is shown in split channels (bottom); scale bars indicate the normalized expression. (I) Boxplots showing enrichment scores for curated adventitial (left) and neuronal (right) gene sets in bFib versus mixFib clusters, showing higher enrichment in bFibs (Wilcoxon test). (J) Confocal immunofluorescence of Jfb placenta stained for CD34 (green) and claudin-5/CLDN5 (red). Left: stitched overview tile scan; right: magnified inset of the boxed region. Scale bars are as indicated. (K) ComplexUpset plot displaying shared and unique differentially expressed genes (DEGs) among bTB-1–bTB-4 clusters. Vertical bars show the number of DEGs for each cluster combination (as defined by connected dots below); horizontal bars show total DEGs per cluster. (L) Gene Ontology enrichment analysis of genes upregulated in bTB clusters relative to human and mouse trophoblasts identifies significant enrichment for pathways involved in nutrient sensing, autophagy, and environmental stress response. Dot size: gene count; color: adjusted *p* value (the key is shown on the right). IF analyses were performed on three independent placentas, with representative images shown.

To contextualize the Jfb placenta within an evolutionary framework, we integrated our bat snRNA-seq data with publicly available datasets from first-trimester human and mid-gestation (E12) mouse placentas (Figure 5A).^20,35,36^ Each dataset contained canonical placental cell types (e.g., TBs, Endos, FBs, and immune cells; Figures S5A-S5D). After dataset-specific quality control, we restricted all data to a shared set of 13,546 genes and standardized gene nomenclature by converting mouse gene symbols to uppercase. Integration and clustering were performed using this harmonized gene set, after which species-specific formatting was restored. The final integrated object contained 110,613 nuclei (bat: 37,278; human: 22,753; and mouse: 50,582) and resolved into 22 clusters (Figure S5F). Clusters composed of >75% nuclei from one species were labeled as bat (BAT), human (HUM), or mouse (MOU), with the remainder labeled as mixed (MIX) (Figures S5F and S5G). This yielded five bat-specific clusters (BAT-1–BAT-5), four human (HUM-1–HUM-4), six mouse (MOU-1–MOU-6), and seven mixed clusters (MIX-1–MIX7). Annotation using canonical markers showed that TB clusters were largely species specific, while endothelial, stromal, and immune cell types frequently overlapped across species (Figures 5B and 5C). Bat-specific clusters included four TBs (bTB-1–bTB-4; BAT-2–BAT-5) and one FB population (bat FB [bFib]; BAT-1). Human-specific clusters were exclusively TBs (hTB-1–hTB-4; HUM-1–HUM-4), while mouse-specific clusters comprised four TBs (mTB-1–mTB-4; MOU-1, MOU-2, MOU-4, and MOU-5), one stromal (MOU-3), and one endothelial (MOU-6). Mixed clusters spanned non-TB types: endothelial (mixEndo-1), FBs (mixed-species FB [mixFib]-1–mixFib-3), Macs (mixMac), stromal cells (mixStroma), and T cells (mixT-cell).

Quantification of species contributions revealed that most TB clusters were highly species restricted, while non-TB clusters showed more overlap (Figure 5D). For example, bTB-3 and bTB-4 were >89% bat derived, and all human TB clusters were >77% human. In contrast, mixEndo-1 and mixMac included nuclei from all species, whereas mixFib-3 lacked bat representation. To explore species-enriched transcriptional programs, we performed differential gene expression using MAST (log_2_FC > 2, min.pct = 0.25), identifying 2,013 bat-specific, 414 human-specific, and 77 mouse-specific genes (Tables S6, S7, and S8). Bat-enriched genes included those involved in growth factor signaling (e.g., *IGF1* and *BMPR1B*) and metabolic functions (*ACE2* and *TYR*). Human- and mouse-specific signatures featured *KISS1* and *KYNU* and *TAF7L* and *FAM135B*, respectively. We then calculated species-specific gene signature scores per cluster, visualized in heatmaps (Figures 5E and 5F). bFibs and TBs (bTB-1–bTB4) showed distinct transcriptional identities, as did human and mouse TBs. Mixed clusters displayed more variable enrichment.

To further characterize bFibs, we examined the top bat-enriched genes and found enrichment for canonical markers of adventitial FBs (e.g., *PIEZO2* and *ZEB1*) and ECM components (*VCAN*, *LAMA2*, etc.; Table S9), suggesting a perivascular identity (Figure 5G). FeaturePlots showed co-expression of *CD34* and *VIM* in bFibs (Figure 5H), and signature scoring confirmed significant enrichment for adventitial markers (Figure 5I). Additionally, bFibs expressed neuron-associated genes (*SOX5*, *PRKG1*, etc.), with high enrichment for a neuronal gene signature (Figure 5I), suggesting a unique FB subtype with dual adventitial and neural features. Confocal staining confirmed the perivascular localization of CD34^+^ FBs near claudin-5^+^ fetal vessels in the labyrinth zone (Figure 5J).

Given the bat-specific signature in TBs, we next identified genes driving this enrichment. Differential expression (DE) analysis (log_2_FC > 2, *padj* < 0.05) across bTB-1–bTB-4 revealed shared and unique transcriptional signatures. ComplexUpset analysis highlighted a conserved bat TB program across all four clusters, with additional subtype-specific enrichments in bTB-1 and bTB-4 (Figure 5K). GO enrichment of bat TB-specific genes revealed the upregulation of pathways related to amino acid starvation responses (*RNF152* and *RRAGD*) and TORC1 regulation, indicating adaptations to nutrient sensing and metabolic stress (Figures 5L and S5J; Table S10). These findings suggest that bat TBs are transcriptionally distinct from human and mouse counterparts, reflecting species-specific placental adaptations.

### Derivation of TB and decidua organoids from the Jfb placenta

**Figure 6.**
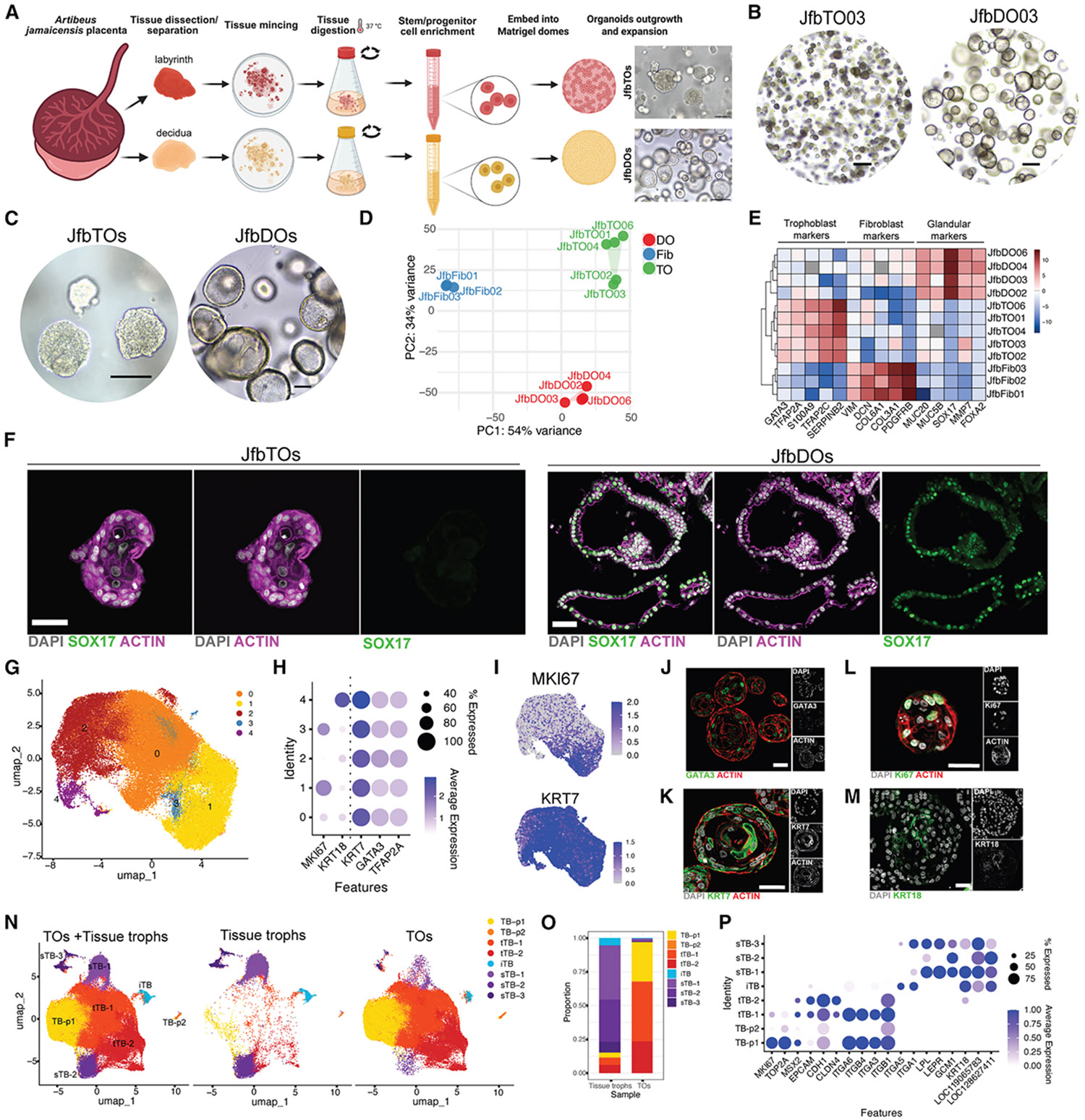
Derivation and characterization of Jamaican fruit bat placenta trophoblast and decidual gland organoids (A) Schematic of Jfb placental organoid derivation. (B) Bright-field images of Jfb trophoblast and decidual organoids (TOs and DOs, respectively). Scale bars, 500 μm. (C) High-magnification bright-field images of established TOs and DOs. Scale bars, 200 μm. (D) Principal-component analysis (PCA) of bulk RNA-seq data showing TOs (green), DOs (red), and fibroblasts (blue) with biological replicates. (E) Heatmap of canonical markers (based on log_2_ RPKM values). (F) Confocal micrographs of JfbTOs (left) or JfbDOs (right) immunostained for SOX17 (green), actin (purple), and DAPI-stained nuclei (blue). Scale bar, 50 μm. (G) UMAP visualization of JfbTOs from snRNA-seq data, resolving five clusters. (H) Dot plot of canonical trophoblast marker expression across TO-derived clusters. The scale is shown on the right. (I) FeaturePlots of *MKI67* (top) and *KRT7* (bottom) expression in TOs. The scales are shown on the right. (J–M) Immunofluorescence staining for GATA3 (J, green), KRT7 (K, green), KI67 (L, green) with actin (red), and KRT18 (M, green). DAPI-stained nuclei (blue); the images on the right show individual grayscale channels. Scale bar, 50 μm. (N) UMAP of integrated JfbTO and subsetted Jfb placenta trophoblast populations from snRNA-seq data, resolving eight clusters. Cluster identities include proliferating trophoblasts, transitional populations, invasive trophoblasts, and syncytiotrophoblasts. (O) Proportional contribution of Jfb placenta trophoblasts and JfbTOs to each cluster. (P) Dot plots of canonical trophoblast markers. The scale is shown on the right. IF analyses were performed on three independent organoid lines, with representative images shown.

The findings above underscore the cellular complexity of the bat placenta and the need for *in vitro* models that recapitulate key TB states. The presence of actively proliferating cells in the Jfb placenta suggested a population of cycling progenitors supporting ongoing TB renewal and that these cells could be isolated and expanded *in vitro* to generate organoids, as shown previously for human, macaque, and pig placentas. To test this, we established TB and decidual gland organoids from Jfb placental tissues using protocols adapted from cross-species organoid derivation.^37,38^ Labyrinth tissue was separated from uterine and decidual compartments, enzymatically digested, and cultured in Matrigel with a defined growth factor cocktail (Figure 6A). This approach yielded two organoid types: TB organoids (JfbTOs) from labyrinth tissue and decidual organoids (JfbDOs) from maternal decidua (Figure 6B), which were successfully established from all five Jfb placentas. Both JfbTOs and JfbDOs grew robustly, forming visible structures within 2 weeks and requiring passaging every 5–7 days. Once established, organoids expanded rapidly following each passage (Figures S6B and S6C). Morphologically, JfbTOs closely resembled human TOs, forming dense, multilayered structures with occasional internal cavities, whereas DOs exhibited cystic morphologies lined by a single epithelial layer (Figures 6B, 6C, S6D, and S6E). To enable quantitative comparison, we developed an image analysis platform to measure the cross-sectional area of all organoids within individual Matrigel domes. This high-throughput approach revealed that JfbTOs were consistently smaller and more compact than human TOs, with significantly reduced cross-sectional areas across replicate cultures (Figures S6E-S6G), reaching a maximal size by 7–8 days postpassaging (Figure S6H).

To confirm the cellular identity and purity of Jfb organoids, we performed bulk RNA-seq on TOs from all five placentas and matched DOs from four, along with primary placental FBs isolated at the time of organoid derivation from three placentas. This design enabled assessment of lineage specificity and exclusion of cross-contamination with FBs or other epithelia. Principal-component analysis (PCA) revealed clear separation among TOs, DOs, and FBs, with tight clustering of biological replicates, demonstrating both distinct cell identities and reproducible organoid generation (Figure 6D). Hierarchical clustering further supported these findings: TOs were enriched for TB markers (e.g., *GATA3*, *TFAP2A*, and *TFAP2C*), DOs expressed glandular epithelial genes (e.g., *MUC20*, *MUC5B*, and *SOX17*), and FBs selectively expressed mesenchymal markers (e.g., *VIM*, *DCN*, and *COL6A1*) (Figure 6E). Differential gene expression analysis using DESeq2 confirmed the strong transcriptional separation of each population (Figure S7A), with volcano plots and heatmaps highlighting lineage-specific gene signatures (Figures S7A and S7B). As an orthogonal validation, single-sample gene set enrichment analysis (ssGSEA) using curated marker sets for TBs, Gland-EpC, and adventitial FBs showed concordant results: TOs scored highest for TB signatures, DOs for Gland-EpC, and FBs for adventitial FB programs (Figure S7C). To visualize lineage specificity *in situ*, we performed confocal imaging for the glandular epithelial marker SOX17, which was robustly expressed in JfbDOs but absent from TOs (Figure 6F). These integrated transcriptomic and imaging analyses confirm the molecular fidelity of each organoid lineage, exclude crosscontamination, and validate the robustness of our organoid derivation platform.

### Defining TB diversity in bat organoids through single-nucleus transcriptomics

To define the cellular composition and differentiation landscape of JfbTOs, we performed snRNA-seq, which generated 62,351 high-quality nuclei that segregated into five transcriptionally distinct TB clusters (Figures 6G and S8A). Clustering was supported by a positive ASW (ASW = 0.191), indicating strong within-cluster cohesion (Figure S8B). Cell cycle scoring identified clusters enriched for S- and G2M-phase genes, consistent with proliferative progenitor populations (Figure S8D). Each cluster expressed canonical TB markers, including *GATA3*, *TFAP2A*, and *KRT7*, as well as distinct marker genes defining specialized states (Figures 6H, 6I, and S8C). Immunostaining confirmed GATA3^+^ and KRT7^+^ cells within JfbTOs (Figures 6J and 6K). Select clusters also expressed MKI67 and KRT18, indicating that TOs encompass a continuum from cycling progenitors to differentiated TBs (Figures 6L, 6M, and S8E).

To place TO-derived clusters in the context of *in vivo* TB diversity, we integrated the TO dataset with our placental TB snRNA-seq dataset (16,174 nuclei; Figure 3). Despite differences in cell number, the integrated UMAP showed extensive mixing between tissue and TO nuclei without dataset-driven segregation (Figure 6N). Integration quality was supported by a near-zero dataset-level ASW (−0.035), indicating a minimal batch effect, and a positive overall ASW (0.121), confirming well-defined cluster structures (Figure S8F). Several TO clusters corresponded to tissue-derived iTB and sTB populations, demonstrating that terminal TB states are recapitulated *in vitro* (Figure 6N). Conversely, TOs were enriched for proliferative and early transitional states, consistent with active progenitor expansion in culture (Figures 6O, S8G, and S8H). Marker-based annotation, guided by canonical TB genes and regulators of intermediate differentiation (e.g., *MSX2*), defined seven major lineages: proliferative (TB-p1/2), transitional (tTB-1/2), invasive (iTB), and syncytial (sTB-1/2/3) (Figure 6O). Altogether, these results show that JfbTOs recapitulate key TB gene expression programs observed *in vivo* while providing access to proliferative and transitional states that are underrepresented in placental tissue.

### Attenuated antiviral responses in bat TOs

**Figure 7.**
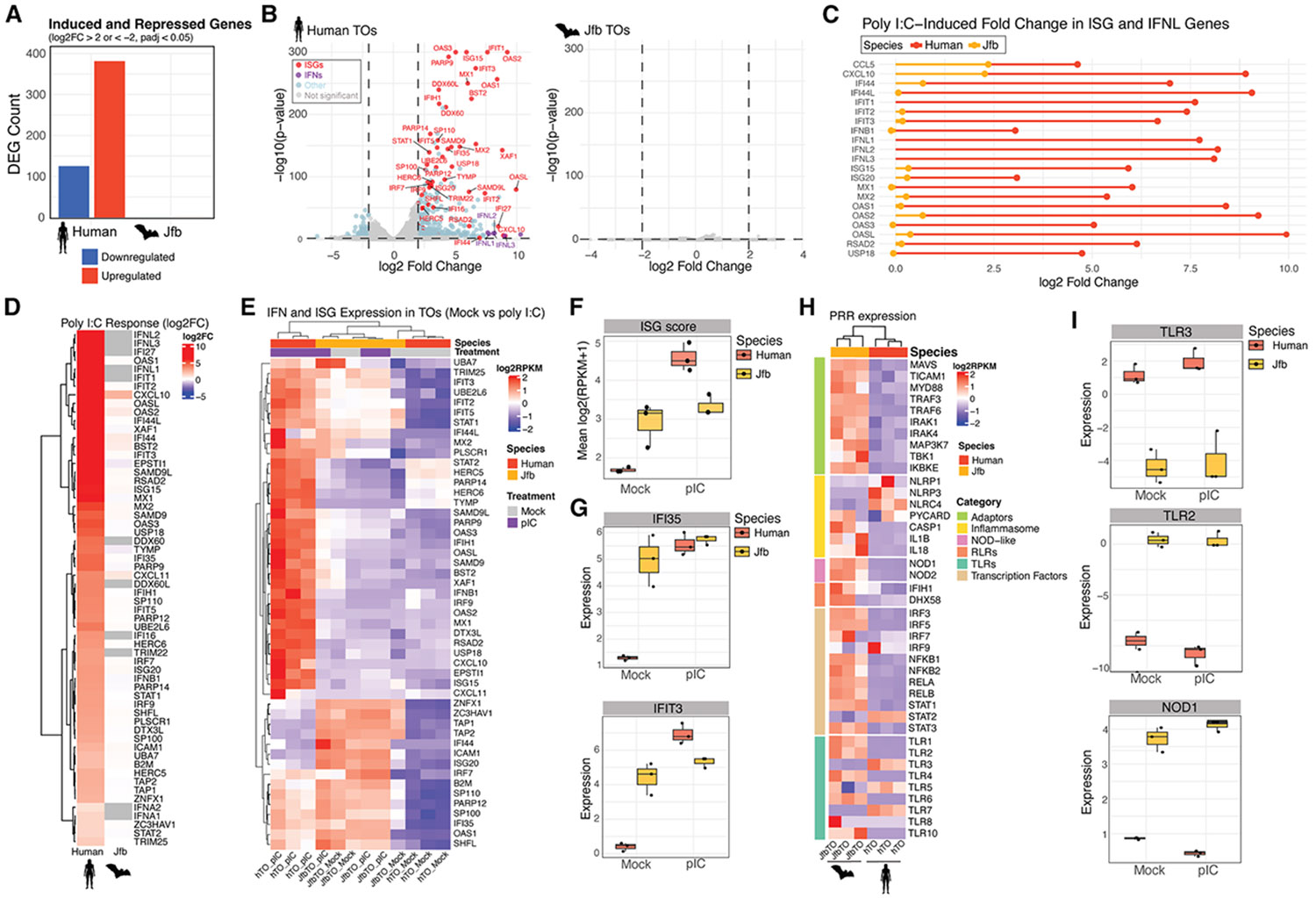
Bat trophoblast organoids exhibit attenuated antiviral signaling compared to those of humans (A) Bar plot showing differentially expressed genes (DEGs) following poly(I:C) treatment in human Tos and JfbTOs, using DESeq2 (log_2_FC > 1, adjusted *p* < 0.05). Upregulated genes are shown in red, and downregulated genes are shown in blue. (B) Volcano plots of DEGs in human (left) and bat (right) TOs after poly(I:C) exposure. Significantly upregulated interferon-stimulated genes (ISGs) are shown in red in human TOs, non-ISG DEGs in light blue, and non-significant genes in gray. (C) Lollipop plot showing poly(I:C)-induced log_2_FC in selected ISGs and interferon lambda (*IFNL*) genes in human (red) and Jfb (yellow) TOs. (D) Heatmap of top poly(I:C)-responsive genes (ranked by log_2_FC) showing markedly higher induction in human TOs compared to JfbTOs. Red indicates high induction, and gray/white indicates no or low induction. The scale is shown on the right. (E) Heatmap showing scaled average expression (*Z* score) of ISGs and IFN genes expressed in mock and poly(I:C)-treated TOs, grouped by species. The scale is shown on the right. (F) Boxplot comparing ISG scores in human TOs and JfbTOs under mock and poly(I:C)-treated conditions. (G) Boxplots showing expression of representative ISGs across species and conditions. (H) Heatmap showing scaled average expression (*Z* score) of key innate immune signaling genes across mock-treated Jfb and human TOs. Genes are grouped by functional category, with color-coded annotations shown on the right. The expression values were derived from gene reads. (I) Boxplots comparing baseline and induced expression of select PRRs. The boxplots in (F), (G), and (I) represent the median (center line), interquartile range (box), and data range within 1.5× the interquartile range. Each point denotes an individual sample, *n* = 3 biological replicates per group. Poly(I:C) experiments were performed using three independent trophoblast organoid lines per species; the plots and heatmaps show representative results.

TOs provide a powerful system for modeling lineage differentiation, hormone secretion, and immune signaling. Given the unique reproductive and immunological traits of bats, we used JfbTOs to define conserved and species-specific antiviral responses. Bulk RNA-seq of human and JfbTOs stimulated with poly(I:C), a synthetic analog of viral double-stranded RNA, revealed a striking contrast. Poly(I:C) induced hundreds of differentially expressed genes (DEGs) in human TOs, including classical interferon-stimulated genes (ISGs) and type III interferon lambda (IFNL) (Figures 7A and 7B; Table S11), but elicited no significant response in JfbTOs (Figure 7B; Table S12). Antiviral ISGs such as *IFI44L*, *IFIT1*, *ISG15*, and *MX1* were strongly induced in human TOs but minimally in JfbTOs (Figures 7C and 7D). Consistent with prior findings,^39^ IFNLs represented the predominant IFNs induced in human TOs (Figure 7C). To assess baseline antiviral readiness, we calculated an ISG score from 30 canonical ISGs. JfbTOs displayed significantly higher basal ISG activity under mock conditions compared to human TOs, which instead showed strong inducibility following stimulation (Figures 7E and 7F). Elevated baseline ISG expression in JfbTOs was also evident in individual genes, such as *IFI35* and *IFIT3* (Figure 7G).

We next examined expression of pattern recognition receptors (PRRs) involved in viral sensing. Human TOs expressed multiple RNA-sensing PRRs, including *TLR3* and *IFIH1*, whereas JfbTOs showed markedly reduced or undetectable expression of these receptors (Figures 7H and 7I). In contrast, bat TOs retained expression of bacterial PRRs, such as *TLR2* and *NOD1*. To test whether this pattern is conserved *in vivo*, we examined PRR expression across TB subtypes in the Jfb placenta using snRNA-seq. Consistent with TOs, sTBs exhibited reduced expression of *TLR3* and *IFIH1*, whereas other subsets maintained higher PRR levels (Figures S9A and S9B). iTBs showed high expression of nearly all TLRs and cytosolic viral sensors, suggesting a distinct immunological profile. Together, these results support a bat-specific strategy in which some TBs maintain elevated basal ISG activity but restrict PRR expression and inducibility in differentiated interface cells, potentially minimizing inflammation while preserving baseline antiviral defense.

## DISCUSSION

Pregnancy in bats occurs under physiological conditions that demand unique adaptations at the maternal-fetal interface. Using single-nucleus transcriptomics, we defined the cellular architecture of the Jfb placenta, identifying diverse TB subtypes and stromal and immune populations with bat-specific transcriptional programs. Specialized Mac subsets expressed pregnancy-associated regulatory molecules linked to tissue remodeling and immune tolerance. Complementary TB and decidua organoid models recapitulated *in vivo* TB states and captured transitional populations otherwise inaccessible in tissue. To facilitate exploration of these findings, we created an interactive ShinyApp encompassing all datasets from placental tissue, TBs, and organoids (https://coynelab.shinyapps.io/bat-placenta-explorer/).

Bats, the second-largest order of mammals, display diversity in placental structure and function. Across Chiroptera, placental architecture ranges from hemochorial to endotheliochorial and epitheliochorial types, reflecting variation in TB invasiveness and maternal remodeling.^3,14^ Here, we provide a high-resolution molecular and cellular atlas of a bat placenta, identifying a bat-specific expansion of PSGs expressed in both TB and Mac populations, an unrecognized feature suggesting novel adaptations in immune regulation and maternal-fetal communication. We did not detect sequences with high similarity to *A. jamaicensis* PSGs in related species (*A. lituratus* and *A. intermedius*), indicating potential lineage specificity. Improved genome annotations across bat taxa will be critical for cross-species comparisons. Broader single-cell and transcriptomic analyses across bat species will further illuminate evolutionary diversification of placental strategies in this mammalian order.

Comparative analysis of placental cell types across bats, humans, and mice revealed species-specific differences, with limited transcriptional overlap among TB populations. A subset of bat sTBs displayed features of an immature intermediate state and appeared to differentiate toward iTBs, defining a lineage trajectory distinct from humans and mice. The Jfb placenta contained a unique iTB population expressing integrins, adhesion molecules, and immunomodulatory factors, paralleling human EVTs. These features likely reflect convergent evolution, as Jfbs sustain prolonged gestations that demand continuous maternal adaptation and immune modulation. Across bat TB lineages, shared transcriptional programs involving TORC1 signaling and cold-stress pathways suggest coordinated metabolic and stress-response adaptations to energy limitation, temperature fluctuation, and oxidative stress during pregnancy.

Stromal FBs also exhibited species-specific adaptations that may meet the unique physiological demands of bat pregnancy. A Jfb-enriched FB population showed a transcriptional profile consistent with adventitial FBs, perivascular stromal cells that provide structural support and mediate immune regulation. Typically positioned at the vessel-tissue interface, these cells integrate mechanical and inflammatory cues to maintain vascular tone and immune homeostasis. In the bat placenta, they appear to assume an expanded role, functioning as structural elements and sensors of infection or stress. Notably, they expressed high levels of PRRs and adaptor molecules (Figure S9A), consistent with innate immune surveillance, along with genes linked to neuronal signaling, suggesting a hybrid identity not observed in other placentas. This dual program may enable them to relay environmental and immunological signals to neighboring TBs and Endos, acting as integrative hubs that coordinate remodeling, vascular responses, and immune signaling.

The immune landscape of the bat placenta revealed adaptations that may enable sustained pregnancy under persistent immune and environmental stress. Placental Macs in the Jfb displayed transcriptional and spatial features of tissue-resident, M2-like cells resembling human Hofbauer cells, which support angiogenesis, tissue remodeling, and immune tolerance. Confocal imaging localized CD163^+^ Macs at the boundary between the labyrinth and iTB zones, positioning them in a regulatory niche where immune, metabolic, and tissue-derived signals converge. Notably, these Macs expressed both a PZP-like gene and a PSG, molecules that are traditionally restricted to TBs or maternal decidual cells in other mammals.^28^ In humans, PZP and PSGs promote maternal tolerance by modulating cytokine responses, supporting regulatory T cell (Treg) differentiation, and limiting inflammation.^40,41^ PZP also acts as a molecular chaperone that stabilizes misfolded proteins, including amyloid beta aggregates linked to preeclampsia and neurode-generation.^42^ Such activity could be particularly advantageous in bats, which experience recurrent hyperthermia and oxidative stress during flight, conditions that favor protein misfolding. Co-expression of PZP-like and PSG genes in Macs suggests that these cells may adopt TB-like immunoregulatory functions. This convergence of TB and Mac gene expression likely represents an evolved strategy to maintain immune tolerance and tissue homeostasis amid chronic microbial exposure and metabolic strain. In parallel, we identified a T cell population exhibiting a spectrum of naive, effector, and regulatory signatures, indicating a dynamic and adaptable immune compartment. Together, these findings reveal a uniquely complex immune niche in the bat placenta, where immune cells appear to adopt hybrid roles that enhance both defense and tolerance, hallmarks of reproductive success in a physiologically extreme context.

Derivation and molecular profiling of Jfb TOs and DOs demonstrated that these models recapitulate key *in vivo* states, including progenitor and differentiated TB lineages. These systems preserve developmental plasticity and permit functional studies of regulatory circuits. JfbTOs exhibited blunted transcriptional responses to poly(I:C) despite high baseline ISG expression, consistent with tonic IFN priming observed in other bat cells.^9,11^ This combination of constitutive readiness and low inducibility may represent a strategy to minimize inflammation while preserving antiviral defense at the maternal-fetal interface. Profiling of PRR expression in bat placentas revealed that viral sensors are selectively downregulated in the STB but retained in iTBs and adventitial FBs, suggesting a redistribution of immune sensing to sentinel populations at tissue boundaries. This lineage- and species-specific architecture may represent a divergent balancing of immune vigilance with tolerance in pregnancy.

Together, these findings define a unique cellular and molecular atlas of the Jfb placenta, uncovering conserved and bat-specific features of TB differentiation, stromal specialization, and immune regulation. By integrating single-nucleus profiling with organoid-based models, we provide a platform for mechanistic studies of bat placentation and a foundation for exploring how evolution repurposes cellular states to support reproduction in physiologically extreme environments.

### Limitations of the study

This work provides a detailed characterization of the *A. jamaicensis* placenta but is limited to a single bat species; therefore, findings may not be generalizable across Chiropteran lineages. Although snRNA-seq enabled detailed investigation of the placental cell state, computational approaches such as clustering and trajectory inference (e.g., Slingshot) rely on parameter choices and infer differentiation from static data, introducing inherent limitations.^43^ Organoid models permitted functional interrogation of bat TB and decidual epithelium but cannot fully recapitulate the multicellular complexity of the native placenta. As a result, immune stimulation experiments performed in organoids may not capture systemic factors or immune-TB crosstalk present *in vivo*. Future *in vivo* work will be important to validate immune response dynamics and mechanisms of tolerance during gestation.

## STAR★METHODS

### EXPERIMENTAL MODEL AND STUDY PARTICIPANT DETAILS

#### Placental tissue collection and processing

Jamaican fruit bats were housed at Colorado State University in a free-flight vivarium under approved IACUC protocols. Animals received fresh fruit, protein and vitamin supplements, and water daily; colony rooms were cleaned biweekly. Visibly pregnant female bats were euthanized, and the entire fetal–placental unit was harvested and photographed. Age of pregnant bats is unknown, and gestational stage was 21–24. Fetal and placental measurements were obtained using ImageJ. Placentas were dissected into four sections and processed for histology (drop-fixed in 10% neutral-buffered formalin), snRNA-seq (flash-frozen), or organoid derivation (stored in Dulbecco’s Modified Eagle Medium on ice). C57BL/6J mice (Jackson Labs #000664) were housed at Duke University under approved IACUC protocols. Ten-week-old females were co-housed with 12-week-old males for 18 days, after which visibly pregnant females were euthanized. Placentas were collected and drop-fixed in 10% neutral-buffered formalin for histological analyses. Tissue code details are provided in Table S13. Placentas were collected from both female and male fetuses, and no sex-specific influences were observed.

### METHOD DETAILS

#### Histology of Jfb and mouse placentas

All staining procedures were performed at HistoWiz, Inc, using the Leica Bond RX automated stainer (Leica Microsystems) and a fully automated workflow. Following fixing in 10% neutral-buffered formalin for 48 h, bat and mouse placentas were shipped to Histowiz in 70% ethanol, embedded in paraffin, and sectioned. Sections were stained with Hemytoxylin & Eosin, Periodic acid–Schiff (PAS) or with antibodies against Pan-Cytokeratin (abcam, ab308262), Vimentin (abcam, ab92547), KRT18 (CK18) (abcam, ab668), CD45 (abcam, ab10558), CD68 (abcam, ab125212), CD3 (abcam, ab16669), F4/80 (Invitrogen, 14-4801-82), Ki67 (abcam, ab155800), or SOX9 (abcam, ab185230). Full-resolution histological images of all stained sections and placental samples are available via HistoWiz using the following links: H&E, PAS, pan-KRT and KRT18, Vimentin, immune cell markers, Ki67, NCAM1, and SOX9.

#### Derivation and culture of trophoblast organoids

Jamaican fruit bat placental tissues were pre-washed and carefully dissected into fetal placental tissue and maternal-derived decidua for isolation. Labyrinth trophoblast stem/progenitor cells were isolated similar to previous protocols.^39^ Briefly, collected labyrinth tissue was cut into small pieces and extensively washed, then sequentially digested with 0.2% trypsin-250 (Alfa Aesar, J63993-09)/0.02% EDTA (Sigma-Aldrich E9884-100G) and 1.0 mg/mL collagenase V (Sigma-Aldrich, C9263-100MG) in small glass containers with stir bars inside placed in a shaking 37° C water bath (Grant Instruments, LSB12US) at 100 rpm. Following collagenase Vdigestion, tissues were manually disrupted by forcefully pipetting up and down about 10 times with a 10 mL serological pipette. Solutions from the two sequential digestions were pooled and filtered through a triple-layered gauze, and the flow-through was collected for downstream processing. The collected flow-through were pooled and centrifuged at 600g for 6 min. The pellet was resuspended with 1 × RBC lysis buffer (Invitrogen, 00433357) for 5 min at room temperature. Pelleted cells were washed once with Advanced DMEM/F12 medium (Life Technologies, 12634-010) and finally resuspended in appropriate volume of ice-cold growthfactor-reduced Matrigel (Corning 356231). Matrigel “domes” (one 40 μl dome/well) were plated into 24-well tissue culture plates (Corning 3526), placed in a 37° C incubator to pre-polymerize for approximately 3 min, turned upside down to ensure equal distribution of the isolated cells in domes for another 10 min, then carefully overlaid with 500 μL of prewarmed full growth media. Jfb trophoblast organoids (TOs) were derived and grown in term trophoblast organoid medium (tTOM) comprised of Advanced DMEM/F12 (Life Technologies, 12634-010) supplemented with 1X B27 (Life Technologies, 17504-044), 1C N2 (Life Technologies, 17502-048), 10% FBS (vol/vol, Cytiva HyClone, SH30070.03), 2 mM GlutaMAX supplement (Life Technologies, 35050-061), 100 μg/mL Primocin (InvivoGen, ant-pm-1), 1.25 mM N-Acetyl-L-cysteine (Sigma, A9165), 500 nM A83-01 (Tocris, 2939), 1.5 μM CHIR99021 (Tocris, 4423), 50 ng/mL recombinant human EGF (Gibco, PHG0314), 80 ng/mL recombinant human R-spondin 1 (R & D systems, 4645-RS-100), 100 ng/mL recombinant human FGF2 (Peprotech, 100-18C), 50 ng/mL recombinant human HGF (Peprotech, 100-39), 10mM nicotinamide (Sigma, N0636-100G), 5 μM Y-27632 (Sigma, Y0503-1MG), and 2.5 μM prostaglandin E2 (PGE2, R & D systems, 22961-0). To passage, JfbTOs were digested using prewarmed TrypLE Express (Gibco, 12605-028) for 8 min in a 37° C shaking water bath at around 190 rpm followed by manual disruption. Disassociated JfbTOs were centrifuged and resuspended with fresh ice-cold growth-factor-reduced Matrigel. To cryopreserve established JfbTO lines, media was removed and Matrigel domes were scraped off and resuspended with CryoStor CS10 stem cell freezing medium (STEMCell Technologies, 07930) and transferred to −80° C for several hours before were deposited into liquid nitrogen tank for long-term storage. Human TOs were cultured with same conditions as JfbTOs.^39^ A table containing the details of organoid codes used in experiments is provided (Table S13).

#### Derivation and culture of decidua gland organoids

Dissected decidua tissues from Jamaican fruit bat placenta were minced into small pieces and washed extensively in wash media (RPMI −1640 with 1x Pen/Strep) prior to being digested in prewarmed dissociation media (1.25 U/mL Dispase II (Sigma-Aldrich, D4693)/0.4mg/mL collagenase V (Sigma-Aldrich, C-9263)) in a 37° C shaking water bath at approximate 100 rpm until decidua glands were observed dominating in the remaining tissues under the microscope (after approximately 20 min initial incubation, checking every 5 min until tissues were digested well as needed). Following digestion, an equal amount of wash media was added, and the remaining tissues were forcefully pipetted approximately 10 times with a 10 mL serological pipette for further dissociation. The released decidual glands were collected by filtration with a 100um-strainer (VWR, 732–2759). The collected glands were pelleted by centrifugation at 600*g* for 6 min and treated with 1 × RBC lysis buffer (Invitrogen, 004333). Following washing with Advanced DMEM/F12 medium, dissociated glands were resuspend in ice-cold growth-factor-reduced Matrigel (Corning 356231), and seeded into Matrigel “domes” in 24-well plates (Corning, 3526), Following polymerization, domes were carefully overlaid with 500 μL prewarmed decidua organoid Expansion Medium (ExM).^44^ ExM was renewed every 2–3 days. Mature decidua organoids were passaged by mechanical disruption following TrypLE Express (ThermoFisher Scientific, 12605010) digestion every 3–5 days. The cryopreservation of established JfbDOs lines was the same as JfbTOs described above.

#### Derivation and culture of primary fibroblast

Primary fibroblast cultures from Jamaican fruit bat placental tissues were established during the initial derivation of JfbTOs. To isolate fibroblasts, the culture media was first aspirated, and cells washed once with 1× DPBS. Then, 1 mL of cell recovery solution (Corning, 354253) was added to each well. Cells were scraped and transferred to microcentrifuge tubes placed on ice to depolymerize the Matrigel.^45^ Once the Matrigel had fully dissolved, the samples were centrifuged at 200 × g for 2 min to pellet the cells. The pellet was washed once with 1× DPBS, then resuspended in DMEM (Corning, 10017CV) supplemented with 10% FBS (Gibco, A56707), 1% penicillin/streptomycin (Gibco, 15140), 1× HyClone non-essential amino acids (Cytiva, SH30238.01), and 1 mM sodium pyruvate (Cytiva, SH30239.01).The entire resuspension from 2 to 3 JfbTO derivation wells was plated into a single well of a 6-well plate. Plates were incubated for 2 h at 37° C to allow fibroblasts to rapidly attach. After 2 h, the plates were gently rocked, and the supernatant was removed to eliminate non-adherent cells. The remaining attached Jfb fibroblasts were maintained in fresh media (as described above).

#### Organoid cross-sectional area analysis

Whole-dome brightfield scans were acquired using a Keyence BZ-X810 all-in-one fluorescence microscope equipped with a motorized XY stage. A 24-well plate was mounted onto the stage, and the BZ-X800 Viewer software was used to control image acquisition. Under “Normal” scan mode, “Capture Still Images” was selected. In the “Observation and Capture” window, the imaging mode was set to “Brightfield.” Using the motorized stage controls, the target dome was located. Both z stack and Stitching functions were enabled in the “Capture Area Setting” menu. The 2× objective lens (PlanApo, NA 0.10) was selected, and four corner points were defined for stitching. z stack limits were set to capture the full depth of the dome, and exposure was adjusted prior to acquisition. Captured image sets were processed using Keyence’s built-in image merging tools to generate stitched, full-focus, high-resolution images of each dome. Organoid cross-sectional area was quantified using Keyence BZ-X800 Analysis Software (v1.1.30.19). From each stitched image, three randomly selected 500 × 500 pixel fields of view per dome were analyzed. A threshold was applied to segment organoids from background, and overlapping organoids were separated using the “Separate Objects” function. Partial organoids at the edges were excluded using “Exclude Objects at Screen Edges,” and debris was removed with the “Remove Objects Less Than” filter (threshold: 200–1500 μm^2^ based on the growth stage of organoids). Organoid counts and cross-sectional areas were calculated per field, and average organoid size was reported per field of view.

#### Sample preparation for immunostaining

Mature JfbTOs and DOs were released from Matrigel domes using established protocols.^45^ Organoids were washed with 1× PBS and fixed in 4% paraformaldehyde (PFA) at room temperature (RT) for 2 h with continuous rotation. Following fixation, organoids were incubated in a 20% (wt/vol) sucrose solution at 4° C overnight to allow them to sink to the bottom of the tube. The next day, the sucrose solution was removed, and approximately 50 μL of 7.5% gelatin/10% sucrose (wt/vol) embedding medium was carefully added. The mixture of organoids and embedding medium was transferred to mini cryomolds (7 × 7 × 5 mm; Simport Scientific, M475) and incubated at 4° C for 20 min to allow polymerization. Samples were then frozen at −80° C for at least 6 h prior to cryosectioning. For Jfb placental tissue, samples were washed with 1× PBS and fixed in 4% paraformaldehyde (PFA) at 4 ° C overnight with continuous rotation. Fixed tissue was then incubated in a 30% (wt/vol) sucrose solution at 4° C overnight. Tissues were transferred into cryomolds (25 × 20 × 5 mm; SAKURA, 4557) for embedding with optimal cutting temperature (OCT) compound and stored at −80° C until sectioning. Frozen organoid blocks and OCT-embedded tissue samples were transferred to a Leica CM1950 cryostat (Leica Biosystems) and equilibrated in the cryochamber at −25° C for 20 min. Tissue blocks were then mounted onto precooled specimen discs using optimal cutting temperature (OCT) compound and placed on the cryostat’s freezing shelf for additional solidification. Once fully stabilized, specimens were secured into the specimen head and sectioned at a thickness of 10–15 μm using a high-profile blade. Cryosections were collected onto charged microscope slides (Fisherbrand, 1255015) and stored at −20° C or processed immediately for downstream staining.

Cryosections were washed with PBS then permeabilized with permeabilization with 0.5% Triton X-100/phosphate-buffered saline [PBS] for 30 min at 4° C.^39,45^ Following permeabilization, organoids were washed and blocked using 5% (v/v) goat serum/0.1% (v/v) Tween 20 in PBS for 15 min at room temperatures. Cryosections are incubated in primary antibodies in the blocking solution described above overnight at 4° C. Then cryosections are washed with PBS and incubated for 1–2 h with secondary antibody at room temperature. Cryosections were washed with PBS and mounted in Vectashield (Vector Laboratories) containing 4′ 6-diamidino-2- phylindole (DAPI).^19,39,45^ The following primary antibodies were used: KRT18 (1:100; Abcam, ab668), pan-KRT (1:100; Abcam, ab308262), KRT7 (1:200; Abcam, ab181598), GATA3 (CST, 5852), E-cadherin (CDH1, Invitrogen, PA5-85088), Vimentin (VIM, Abcam, 1:250 ab137321), NCAM-1 (1:200; Abcam, ab9018), CD34 (1:200; Abcam, ab81289), Claudin-5 (1:100; Invitrogen 35–2500), CD163 (1:250; Abcam ab182422), and SOX17 (1:500, Abcam, Ab224637). Secondary antibodies included Multi-rAb CoraLite Plus 488 Goat anti-mouse (Proteintech, RGAM002), Multi-rAb CoraLite Plus 594 Goat anti-rabbit (Proteintech, RGAR004), goat anti-mouse Alexa Fluor 488 and 594 (Invitrogen, A-11001 and A-11005), and goat anti-rabbit Alexa Fluor 488 and 594 (Invitrogen, A-11008 and A-11012). Phalloidin conjugates used were Alexa Fluor 594–phalloidin (Invitrogen, A12381) and Alexa Fluor 647–phalloidin (Invitrogen, A22287). Confocal images were acquired on an Olympus FLUOVIEW FV3000RS (IX83 inverted; hybrid resonant/galvo; 405/488/561/640 nm) with TruSpectral detection (two cooled GaAsP + two PMT). Acquisition (including tile-stitching) was performed in the FLUOVIEW control software (FV31S, Advanced module). Images were collected using 10× UPLXAPO (NA 0.40, air), 20× UPLXAPO (NA 0.80, air), and 40× UPLSAPO40XS (NA 1.25, silicone oil) objectives. Image contrast was adjusted using Fiji (v2.14.0/1.54f) or Adobe Photoshop (v24.3.0). In some cases, pseudocoloring was applied for optimal visualization using Fiji or Fluoview software. Image analysis and 3D reconstructions were performed using FIJI.

#### Whole-mount immunofluorescence staining

Mature trophoblast organoids were released and collected for fixation as described above. Instead of embedding for cryosectioning, intact organoids were processed directly for whole-mount immunostaining following permeabilization as described above with cryosections.^39,45^ All centrifugation steps were replaced by gravity sedimentation to preserve organoid morphology. To prevent compression during mounting, small dots of Vaseline were applied to the corners of each coverslip. z stack fluorescent images were acquired using an Olympus Fluoview FV3000 inverted confocal microscope.

#### AlphaFold3 structural prediction

Predicted protein structures for bat, human, and mouse pregnancy-specific glycoproteins (PSGs) were generated using AlphaFold3.0. Structural overlays were performed using ChimeraX (version 1.9)^46^ to align predicted models based on backbone RMSD minimization. Structures were visualized and colored by species for comparison, and movies were generated within ChimeraX to highlight domain conservation and overall architectural similarity across species.

#### RNA extraction and bulk RNAseq

Total RNA was extracted from established JfbTOs, JfbDOs, and fibroblasts (Jfb Fibroblasts) at early passage (see Table S13) with Sigma GenElute Universal total RNA purification kit (Sigma-Aldrich, RNB100) following manufacturer’s instruction. Purified Total RNA concentration and quality was determined by Thermo scientific Nanodrop One spectrophotomer. All total RNA samples submitted for bulk RNA-seq were further run for QC evaluation for their RQN (RNA quality number, >7 for all samples) prior to library preparation by the Duke Sequencing and Genomic Technologies (SGT) using KAPA HyperPrep kit (Roche). Libraries were sequenced on the NovaSeq X Plus 10B lane. The reads were aligned to the Jfb (*Artibeus jamaicensis*) genome assembly (GCF_021234435.1_CSHL_Jam_final_genomic.fna) using the Rsubread package (v2.10.0) in R version 4.1.^47^ Alignment indices were first generated with *buildindex()*, and paired-end reads were aligned using *align()* with four threads. Gene-level quantification was performed using *featureCounts()* (Rsubread) with the associated genome annotation file (GCF_021234435.1, GTF format). Genes were counted based on exon features, and counts were generated for each sample. To facilitate downstream normalization, reads per kilobase per million mapped reads (RPKM) values were calculated for each sample, using gene lengths extracted from the annotation file and the total number of mapped reads per sample. Following quantification, differential expression analysis was performed using the DESeq2 package (v1.34.0) in R.^48^ Principal Component Analysis (PCA) was conducted using the *prcomp()* function to visualize major sources of variance between conditions. Heatmaps of log_2_-transformed RPKM values were generated with the pheatmap package (v1.0.12) in R.^49^ Volcano plots highlighting differentially expressed genes were created using GraphPad Prism (version 9.0). Files associated with bulk RNA-seq studies have been deposited into Sequence Read Archive (PRJNA1251670).

To quantify enrichment of lineage-specific transcriptional programs, we performed single-sample gene set enrichment analysis (ssGSEA) on bulk RNA-seq data from TOs, DOs, and fibroblast cultures. Raw counts were normalized using the DESeq2 package with rlog transformation. Curated gene sets were compiled for adventitial fibroblasts (e.g., *PDGFRA*, *PIEZO2*, *IGF1*), trophoblasts (e.g., *GATA3*, *TFAP2C*, *KRT7*), and glandular epithelial cells (e.g., *PRL*, *LRP2*, *SOX17*). ssGSEA was implemented using the GSVA package, and scores were compared across sample types using one-way ANOVA.

Jfb and human TOs were treated with 10 μg/mL high molecular weight poly I:C (Invivogen, tlrl-pic) or mock-treated for ~24 h. Total RNA was extracted as described above. RNA-seq libraries were prepared using the Watchmaker mRNA library prep kit and sequenced on an Illumina NovaSeq X Plus platform (~70 million 50bp paired-end reads per sample). Reads were aligned to the *Artibeus jamaicensis* or GRCh38 genome using STAR, and gene-level counts were generated with featureCounts. Differential gene expression was analyzed using DESeq2. Technical replicates were grouped by biological replicate (Code) and modeled with a paired design (~ Code + Treatment). Differentially expressed genes were identified by comparing poly I:C–treated versus mock-treated TOs, with significance defined as adjusted *p*-value <0.05.

#### Processing for single-nuclei RNA sequencing

To create a single cell suspension of organoids, mature trophoblast organoids were collected at early passage (see Table S13) and incubated with TrypLE Express (Invitrogen, 12605036) in a 37° C shaking water bath at approximately 190 rpm for 15 min. Organoids were pelleted by centrifugation at 1250 rpm for 3 min and resuspended with 200μL Advanced DMEM/F12 supplemented with 2 mM GlutaMAX supplement, 10 mM HEPES (Gibco, 15630-106), and 1 × Penicillin/Streptomycin (Lonza, 17-602E) (basal media). Then organoids were manually disrupted using a single channel p200 pipette ~200 times followed by the addition of 800 μL basal media. Dissociated organoids were pelleted by centrifugation at 1250rpm for 5 min and the pellet was resuspended in approximately 250 μL of 1x PBS containing 1% FBS. Sequencing was performed on four organoid lines from unique placental tissues.

For single nuclei RNA-sequencing, flash frozen placentas, with uterus removed, or JfbTOs dissociated as described above were subjected to RNA QC and nuclear isolation using 10x Genomics’ Nuclei Isolation protocol (10x Genomics – Pleasanton, VA) per the manufacturer’s instructions. RNA quality was assessed using an Agilent 4200 TapeStation System. Four placental samples and four organoid samples (see Table S13) had an RNA integrity score (RIN) > 7 and were used for downstream nuclear isolation. To isolate nuclei, 10mg of minced frozen tissue or a single dome of organoids dissociated into single cells, were resuspended in 0.11x lysis buffer (275 μL Lysis Buffer +0.275 μL Reducing Agent B + 2.75 μL Surfactant +2502.23 μL 1x chilled PBS) and homogenized using a pestle (approximately 60-70x), prior to incubation on ice for 3–5 min. Homogenized samples were then washed twice with nuclei wash and resuspension buffer, followed by centrifugation at 500rcf, 4° C, for 10 min. Supernatants were then removed, and nuclei resuspended in appropriate volume of wash and resuspension buffer to a concentration of approximately 1.5 × 10^5^ nuclei/mL, which was determined on a Cellometer Ascend Automated Cell Counter (RevvityWaltham, MA). If necessary, nuclei were filtered through 40μm Flowmi Cell Strainers (Bel-Art – Wayne, NJ) to remove clumps and debris prior to proceeding with the single nuclei assay. Nuclei were visualized at 40× magnification on a LifeTech EVOS FL microscope (Thermo Fisher Scientific – Waltham, MA) to determine quality via nuclear membrane intactness. All samples examined in this study had a nuclear intactness ≥80% and a viability <1% to ensure high quality nuclear isolations. 20,000 single nuclei of each sample were loaded with a Chromium Reagent Kit v4. Tissue libraries were sequenced on a 10B flow cell of the Nova Seq X Plus at a targeted depth of 70,000 reads per nucleus. Cellranger was then used to align reads to the Jamaican Fruit Bat genome (GCF_021234435.1) and create a counts matrix. Files associated with snRNA-seq studies have been deposited into Sequence Read Archive (PRJNA1251203 and PRJNA1251235).

#### snRNA sequencing data processing and integration

To analyze snRNA-seq data from placental tissue and TOs, we first processed and merged individual datasets separately by sample type. Mitochondrial gene expression was assessed using a curated list of mitochondrial genes (*ND5*, *ND4*, *ND3*, *ND1*, *ND2*, *ND6*, *ND4L*, *CYTB*, *COX2*, *ATP8*, *ATP6*, *COX3*, *COX1*), and mitochondrial and ribosomal RNA content were calculated using the *PercentageFeatureSet()* function. Quality control filtering was tailored to each dataset: for placenta tissue, we retained nuclei with 300–9000 detected genes, fewer than 20,000 UMI counts, ribosomal RNA content below 1.2%, and mitochondrial RNA content below 1.5%. For TOs, dataset-specific thresholds were applied, generally retaining nuclei with 300–9000 genes, fewer than 40,000 UMI counts, ribosomal RNA content below 9%, and mitochondrial RNA content below 2%.

All analyses were conducted in R version 4.3.2 using Seurat v5.0.1, SeuratWrappers v0.3.0 for Harmony integration, and MAST v1.26.0. For figure generation and analysis, ComplexUpset v1.3.3 and ggplot2 v3.5.0 were also used. Sequencing data were aligned and quantified using Cell Ranger v6.1.2 (10x Genomics) against the Jamaican fruit bat genome (annotation release 100), GRCh38 for human, and GRCm39 for mouse. Following quality control, data normalization and dimensionality reduction were performed using the Seurat v5 pipeline. Layers within each dataset were merged using *JoinLayers()* and split by sample identity. We applied *SCTransform()* to normalize gene expression and regress out unwanted technical variation, including gene count, UMI count, and the percentages of mitochondrial and ribosomal RNA. Dimensionality reduction was performed with principal component analysis (PCA) using *RunPCA(),* and the number of components used for downstream analysis was determined using an elbow plot *(ElbowPlot()).* To integrate data across samples while correcting for batch effects, we used Harmony-based integration for placenta tissue (IntegrateLayers() with HarmonyIntegration()) and canonical correlation analysis (CCA) for TOs. In both cases, PCA was replaced with a batch-corrected low-dimensional space for downstream analysis. Clustering was performed using *FindNeighbors()* and *FindClusters()* with a resolution of 0.8 for placenta and 0.125 for TOs. Visualization using Clustree() and marker gene enrichment were utilized to determine clustering resolution that reflects biological populations. Higher resolution was used for tissue to resolve interstitial and immune subpopulations, while a lower resolution was chosen for organoids to prevent over-clustering of compact trophoblast lineages. Batch-corrected UMAP embeddings were generated using *RunUMAP()* to visualize the final integrated structure of each dataset. To assess lineage relationships among trophoblast clusters, we applied *Slingshot* trajectory inference^50^ using PCA embeddings as input as input and designated the proliferating trophoblast cluster as the root. This enabled reconstruction of putative differentiation trajectories based on transcriptomic similarity, revealing transitions from progenitor to differentiated states. To quantitatively assess cluster validity, silhouette scores were computed based on distances in the selected dimensionality reduction space (pca or umap). Silhouette distributions were examined per cluster and compared to randomized cluster assignments to evaluate structure relative to noise. This approach provided an additional quantitative validation step to confirm that clusters reflected true biological differences rather than technical variation or overfitting.

Differential gene expression analysis was conducted using *FindAllMarkers()* on the normalized and scaled RNA assay. Marker genes were defined using a minimum log fold change threshold of 0.25 and required expression in at least 25% of nuclei within a cluster (min.pct = 0.25). Marker genes were identified separately for placenta and TO datasets. To address sparsity in gene expression, we applied Adaptively-thresholded Low-Rank Approximation (ALRA) using *RunALRA()* on the SCT assay to impute missing values. The processed and integrated placenta and TO datasets were retained for all downstream analyses.

To assess enrichment of lineage-specific programs in fibroblast populations from the single-nucleus RNA-seq dataset, we computed per-cell signature scores for adventitial fibroblasts and neuronal-associated gene sets. A curated adventitial signature (e.g., *PDGFRA, PIEZO2, IGF1*) and a neuronal-like signature (e.g., *PRICKLE1, MAPK10, GPC4*) were assembled based on published markers and observed gene enrichments in bat-specific fibroblasts (bFib). Signature scores were calculated using Seurat’s *AddModuleScore()* function and aggregated by fibroblast cluster. Score distributions were visualized using boxplots, and statistical comparisons were performed using Wilcoxon rank-sum tests. Full gene lists are provided in Table S9. To evaluate proliferative states, we calculated cell cycle scores using Seurat’s *CellCycleScoring()* function with curated gene sets for S-phase and G2/M-phase markers. This enabled identification of actively cycling cells across both tissue and organoid datasets and supported annotation of trophoblast progenitor populations.

Differential gene expression analysis was performed using the MAST framework to compare each bat trophoblast cluster (bTB-1 through bTB-4) against human and mouse trophoblasts. Genes were considered significantly upregulated if they met a log_2_ foldchange >2 and adjusted *p*-value <0.05. To visualize shared and cluster-specific differentially expressed genes, we generated an intersection plot using the ComplexUpset R package. A binary matrix of gene-cluster associations was constructed, and intersections across clusters were visualized, highlighting the conserved transcriptional program shared among bat trophoblast populations.

Custom code used for data analysis and figure generation is available at CoyneLabDuke GitHub repository.

#### Cross-species snRNA sequencing analysis

To enable integrated analysis across species, we processed snRNA-seq datasets from Jfb placenta, first-trimester human placenta, and mid-gestation (E12) mouse placenta using a standardized workflow. We utilized publicly available snRNA-seq datasets from human (PRJNA1035951)^20^ and mouse placenta PRJNA954811, PRJNA781579).^35,36^ Because gene symbols differ in case formatting across species, we standardized mouse gene names to uppercase to match bat and human gene naming conventions. This was done by extracting the RNA assay count matrix from the mouse Seurat object, converting all gene names to uppercase using toupper(), and removing version suffixes (e.g., “.1”, “.2”) using regular expressions. To avoid duplicated gene names after case conversion, a custom function was applied to append unique identifiers (e.g., “_DUP1”, “_DUP2”) to duplicated entries. The modified count matrix was then used to generate a new Seurat object with standardized gene names, and UMAP embeddings, cluster identities, and metadata from the original mouse object were transferred to preserve downstream analyses. RNA and ALRA assays were also recreated in the renamed object to ensure consistency across layers.

For integration, shared genes were identified across all three species by computing pairwise intersections of RNA assay gene sets using *LayerData()* and *intersect()* (base R). This resulted in a set of 13,546 genes shared across bat, human, and mouse datasets. Each object was subset to retain only these genes, ensuring compatibility across species. Normalization was performed independently for each dataset using *SCTransform()* v2 in Seurat v5, with regression of covariates including gene count, UMI count, and mitochondrial and ribosomal RNA percentages. Normalized data were stored in new SCT assay layers. The normalized datasets were then merged using *merge()* to form a unified Seurat object. Dimensionality reduction was performed using PCA (*RunPCA()),* and integration was carried out using *IntegrateLayers()* with HarmonyIntegration, using species identity as the batch variable. The Harmony-reduced space was used for neighbor detection (*FindNeighbors()),* clustering (*FindClusters(),* resolution = 0.3), and visualization (*RunUMAP()),* enabling integrated cross-species analysis of conserved and divergent placental cell types.

To preserve species-specific transcriptomic complexity, Harmony and UMAP embeddings from the integrated object were projected back onto the original, full-genome Seurat objects using *Embeddings()* and *CreateDimReducObject()* with species-specific assay settings. Only shared cells between the original and integrated datasets were retained. Harmony embeddings were added to the SCT assay, and UMAP coordinates to the RNA assay, using distinct keys (“harmony_”, “UMAPharmony_”). Each dataset was then independently clustered using Harmony reduction and FindClusters() (resolution = 0.3), allowing for visualization and downstream analysis of integrated cell states while retaining the full gene expression profile of each species.

To annotate predicted genes labeled as “LOC” identifiers in the *Artibeus jamaicensis* genome, we developed a custom R-based pipeline. LOC IDs were queried against the NCBI Gene database using the rentrez R package^51^ to retrieve corresponding official gene names, functional descriptions, and gene types. LOC IDs were queried against the NCBI Gene database using the rentrez R package^51^ to retrieve corresponding official gene names, functional descriptions, and gene types. For each LOC ID, we first retrieved the associated NCBI Gene ID, then extracted the gene name, description, and annotation status (e.g., validated, provisional). The resulting annotated gene list was merged with the original LOC dataset and exported for downstream analysis.

#### Recipes

**Table:** 

Basal media		
Reagent	Final concentration	Amount
Penicillin-streptomycin (100 X)	1 X	1 mL
HEPES (100 X)	1 X	1 mL
L-glutamine (100 X)	1 X	1 mL
Advanced DMEM/F12	N/A	97 mL
Total	N/A	100 mL

*Note:* The prepared media can be stored at 4° C for up to 1 month, pre-warm the media prior to every time use.

**Table:** 

Jfb placenta tissue dissociation mediaIfor to derivation	
Components	Final concentration (g/L)
Glucose	0.3
NaCl	12
KCl	0.3
Na_2_HPO_4_	1.725
KH_2_PO_4_	0.3
Trypsin-1:250 (Thermo scientific, J63993.18)	2
EDTA	0.2

*Note:* Dissolve above items into 1 L of water, filter solution for sterilization, then aliquot for one-time use and store in −20° C for up to 1 year.

**Table:** 

Jfb placenta tissue dissociation media II for to derivation	
Components	Final concentration
Collagenase V (STEMCELL Technologies, 100–0681)	1 mg/mL
FBS (Gibco, 26140-079)	10% (vol/vol)
Ham F-12 (Gibco, 11765-047)	N/A

*Note:* make above media fresh for each isolation.

**Table:** 

Jfb placental decidua dissociation media for do derivation	
Components	Final concentration
Collagenase V (STEMCELL Technologies, 100–0681)	400 mg/L
Dispase II (Sigma, D4693)	1.25 g/L
FBS (Gibco, 26140-079)	10% (vol/vol)
RPMI-1640 (Cytiva, SH30027.02)	N/A

Note: make above media fresh for each isolation.

**Table:** 

tTOM for Jfb to culture		
Reagent	Final concentration	Amount (μL)
N2 (100 X)	1 X	500
B27 (50 X)	1 X	1000
Primocin (500 X)	100 μg/mL	100
NAC (80 X)	1.25 mM	625
L-glutamine (100 X)	2 mM	500
A83-01 (10000 X)	500 nM	5
CHIR99021 (10000 X)	1.5 μM	5
recombinant human EGF (2000 X)	50 ng/mL	25
recombinant human R-spondin1 (2000 X)	80 ng/mL	25
recombinant human FGF2 (2000 X)	100 ng/mL	25
recombinant human HGF (2000 X)	50 ng/mL	25
Nicotinamide (100 X)	10 mM	500
Y-27632 (200 X)	5 μM	250
PGE2 (2000 X)	2.5 μM	25
FBS	10% (v/v)	5000
Advanced DMEM/F12	N/A	adjust volume to 50 mL
Total	N/A	50 mL

**Table:** 

ExM for Jfb do culture		
Reagent	Final concentration	Amount (μL)
N2 (100 X)	1 X	500
B27 (50 X)	1 X	1000
Primocin (500 X)	100 μg/mL	100
NAC (80 X)	1.25 mM	625
L-glutamine (100 X)	2 mM	500
A83-01 (10000 X)	500 nM	5
recombinant human EGF (2000 X)	50 ng/mL	25
recombinant human Noggin (1000 X)	100 ng/mL	50
recombinant human R-spondin1 (2000 X)	80 ng/mL	25
recombinant human FGF10 (1000 X)	100 ng/mL	25
recombinant human HGF (2000 X)	50 ng/mL	25
Nicotinamide (100 X)	10 mM	500
Advanced DMEM/F12	N/A	adjust volume to 50 mL
Total	N/A	50 mL

*Note:* The prepared media can be stored at 4° C for up to 2 weeks, pre-warm the media prior to every time use.

### QUANTIFICATION AND STATISTICAL ANALYSIS

All experiments conducted within this study were reproduced using independent samples including tissues and organoids (see Table S13 for details). Statistical significance was defined as described in figure legends. For all statistical tests determining significance, *p* value < 0.05 was defined as statistically significant.

## Supplementary Material

Supplemental materials

Supplemental information can be found online at https://doi.org/10.1016/j.celrep.2025.116645.

## Figures and Tables

**Table:** KEY RESOURCES TABLE

REAGENT or RESOURCE	SOURCE	IDENTIFIER
Antibodies		
Pan-Cytokeratin (pan-KRT)	Abcam	ab308262;RRID:AB_3676255
Vimentin	Abcam	ab92547;RRID:AB_10562134
KRT18 (CK18)	Abcam	ab668;RRID:AB_305647
CD45	Abcam	ab10558;RRID:AB_442810
CD68	Abcam	ab125212;RRID:AB_10975465
CD3	Abcam	ab16669;RRID:AB_443425
F4/80	Invitrogen	14-4801-82;RRID:AB_467558
Ki67	Abcam	ab15580;RRID:AB_443209
SOX9	Abcam	ab185230;RRID:AB_2715497
KRT7	Abcam	ab181598;RRID:AB_2783822
GATA3	Cell Signaling Technology (CST)	5852;RRID:AB_10835690
NCAM-1	Abcam	ab9018;RRID:AB_306945
CD34	Abcam	ab81289;RRID:AB_1640331
Claudin-5	Invitrogen	35-2500;RRID:AB_87321
CD163	Abcam	ab182422;RRID:AB_2753196
SOX17	Abcam	Ab224637;RRID:AB_2801385
Goat anti-mouse IgG, CoraLite Plus 488 (Multi-rAb)	Proteintech	RGAM002;RRID:AB_3068538
Goat anti-rabbit IgG, CoraLite Plus 594 (Multi-rAb)	Proteintech	RGAR004;RRID:AB_3073502
Goat anti-mouse IgG, Alexa Fluor 488	Invitrogen	A-11001;RRID:AB_2534069
Goat anti-mouse IgG, Alexa Fluor 594	Invitrogen	A-11005;RRID:AB_2534073
Goat anti-rabbit IgG, Alexa Fluor 488	Invitrogen	A-11008;RRID:AB_143165
Goat anti-rabbit IgG, Alexa Fluor 694	Invitrogen	A-11012;RRID:AB_2534079
Phalloidin, Alexa Fluor 594	Invitrogen	A12381
Phalloidin, Alexa Fluor 647	Invitrogen	A22287
Biological samples		
Jamaican fruit bat placentas (pregnant females)	Jamaican fruit bat placentas (pregnant females)	See Table S12
Mouse placentas (C57BL/6J)	Mouse placentas (C57BL/6J)	The Jackson Laboratory #000664
Human Trophoblast Organoids	Cryopreserved samples organoid lines from previous studies	Yang et al.^39^
Chemicals, peptides, and recombinant proteins		
Trypsin-250 (0.2%)	Alfa Aesar	J63993-09
EDTA (0.02%)	Sigma-Aldrich	E9884-100G
Collagenase V (1.0 mg/mL)	Sigma-Aldrich	C9263-100MG
Dispase II (1.25 U/mL)	Sigma-Aldrich	D4693
Collagenase V (0.4 mg/mL)	Sigma-Aldrich	C-9263
RBC Lysis Buffer (1×)	Invitrogen	00433357
Advanced DMEM/F12	Life Technologies	12634-010
Growth Factor Reduced Matrigel	Corning	356231
24-well tissue culture plate	Corning	3526
B27 Supplement (1×)	Life Technologies	17504-044
N2 Supplement (1×)	Life Technologies	17502-048
Fetal Bovine Serum (FBS), 10%	Cytiva HyClone	SH30070.03
GlutaMAX Supplement, 2 mM	Life Technologies	35050-061
Primocin, 100 μg/mL	InvivoGen	ant-pm-1
N-Acetyl-L-cysteine, 1.25 mM	Sigma	A9165
A83-01, 500 nM	Tocris	2939
CHIR99021, 1.5 μM	Tocris	4423
EGF, recombinant human, 50 ng/mL	Gibco	PHG0314
R-spondin 1, recombinant human, 80 ng/mL	R&D Systems	4645-RS-100
Noggin, recombinant human, 100 ng/mL	Peprotech	120-10C
FGF2, recombinant human, 100 ng/mL	Peprotech	100-18C
FGF10, recombinant human, 100 ng/mL	Peprotech	100-26
HGF, recombinant human, 50 ng/mL	Peprotech	100-39
Nicotinamide, 10 mM	Sigma	N0636-100G
Y-27632, 5 μM	Sigma	Y0503-1MG
Prostaglandin E2 (PGE2), 2.5 μM	R&D Systems	22-961-0
TrypLE Express	Gibco	12605-028
TrypLE Express	ThermoFisher Scientific	12605010
Cell Recovery Solution	Corning	354253
DMEM (fibroblast media)	Corning	10017CV
FBS (for fibroblast media)	Gibco	A56707
Penicillin/Streptomycin (1×)	Gibco	15140
Non-essential amino acids (NEAA), 1×	Cytiva	SH30238.01
Sodium Pyruvate, 1 mM	Cytiva	SH30239.01
HEPES, 10 mM	Gibco	15630-106
Penicillin/Streptomycin (Lonza)	Lonza	17-602E
100 μm Cell Strainer	VWR	732-2759
Mini cryomolds (7 × 7 × 5 mm)	Simport Scientific	M475
Microscope slides	Fisherbrand	1255015
Flowmi Cell Strainers, 40 μm	Bel-Art	H13680-0040
Deposited data		
snRNA-seq datasets	This study	PRJNA1251203; PRJNA1251235
Bulk RNA-seq (poly I:C vs. mock)	This study	PRJNA1251670
Publicly available datasets used in this study		
Human first-trimester snRNA-Seq dataset	Wang et al.^20^	PRJNA1035951
Mouse snRNA-Seq dataset 1	Lopez-Tello et al.^35^	PRJNA954811
Mouse snRNA-Seq dataset 2	Guo et al.^36^	PRJNA781579
Critical commercial assays and instruments		
Keyence BZ-X810 microscope + motorized XY	Keyence	BZ-X810
Keyence BZ-X800 Analysis Software	Keyence	v1.1.30.19
Olympus Fluoview 3000 inverted confocal	Olympus	FV3000
EVOS FL fluorescence microscope	Thermo Fisher Scientific	EVOS FL
Grant shaking water bath	Grant Instruments	LSB12US
Agilent TapeStation System	Agilent	4200
Cellometer Ascend Automated Cell Counter	Revvity	Ascend
Chromium Reagent Kit v4	10x Genomics	v4 (Kit)
NovaSeq X Plus	Illumina	Instrument
Software and algorithms		
Keyence BZ-X800 Analysis (v1.1.30.19)	N/A	https://www.keyence.com/landing/microscope/lp_fluorescence.jsp
Cell Ranger	10x Genomics	v6.1.2
Seurat	Satija Lab	v5.0.1
SeuratWrappers (Harmony integration)	Satija Lab	v0.3.0
MAST	Bioconductor	v1.26.0
ComplexUpset	CRAN	v1.3.3
ggplot2	CRAN	v3.5.0
STAR	Dobin Lab	N/A
featureCounts (Rsubread)	Bioconductor	v2.10.0
DESeq2	Bioconductor	v1.34.0
FIJI/ImageJ	NIH	v2.14.0/1.54f
ChimeraX	UCSF	v1.9
Custom Code	This paper	10.5281/zenodo.17289723

## Highlights

- snRNA-seq defines cell types and differentiation pathways in the bat placenta
- Bat placental organoids reflect *in vivo* cell signatures and lineage trajectories
- Transcriptional programs revealed in bat trophoblast, stromal, and immune lineages
- Bat trophoblasts show attenuated antiviral responses compared to human counterparts
