# Supplementary material for: Cellular and immune adaptations at the maternal-fetal interface in bats
Source: Cell Rep. Author manuscript; Available in PMC 2026 Apr 16. (PMC13084540; doi:10.1016/j.celrep.2025.116645)
Supplement: Supplemental materials [file NIHMS2160197-supplement-Supplemental_materials.zip › 1-s2.0-S2211124725014172-mmc1.pdf]

Cell Reports, Volume *44*

Supplemental information

Cellular and immune adaptations

at the maternal-fetal interface in bats

Allyson Caldwell, Liheng Yang, Rebecca L. Casazza, Rizban E. Worota, Cole McCutcheon, Patrick S. Creisher, Erika Zhan, Clara Reasoner, Ashley Higgins, Tony Schountz, and Carolyn B. Coyne

Figure S1

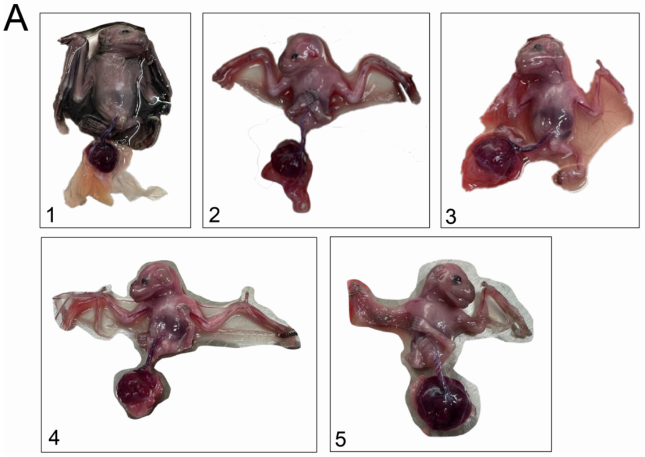

B

Bat ID #	Placenta Length (cm)	Forelimb Length	CRL (cm)	CRL: Plac ratio	CRL: forelimb ratio	Notable developmental features	Estimated gestational stage	Sex
1	1.48	3.20	5.46	3.69	1.70	Open eyes, skin pigment and wrinkling, claw pigment, wings opaque, nose-leaf evident.	24+ (fetal period)	Female
2	1.45	1.91	3.89	2.68	2.04	Open eyes, claw pigment, nose-leaf evident, wings translucent.	21-23	Male
3	1.60	1.46	4.12	2.57	2.82	Open eyes, claw pigment, wings opaque, nose-leaf evident, wings translucent	21-23	Female
4	1.55	1.43	3.70	2.39	2.59	Open eyes, claw pigment, wings opaque, nose-leaf evident, wings translucent	21-23	Male
6	1.83	1.75	3.98	2.18	2.27	Open eyes, claw pigment, wings opaque, nose-leaf evident, wings translucent	21-23	Male

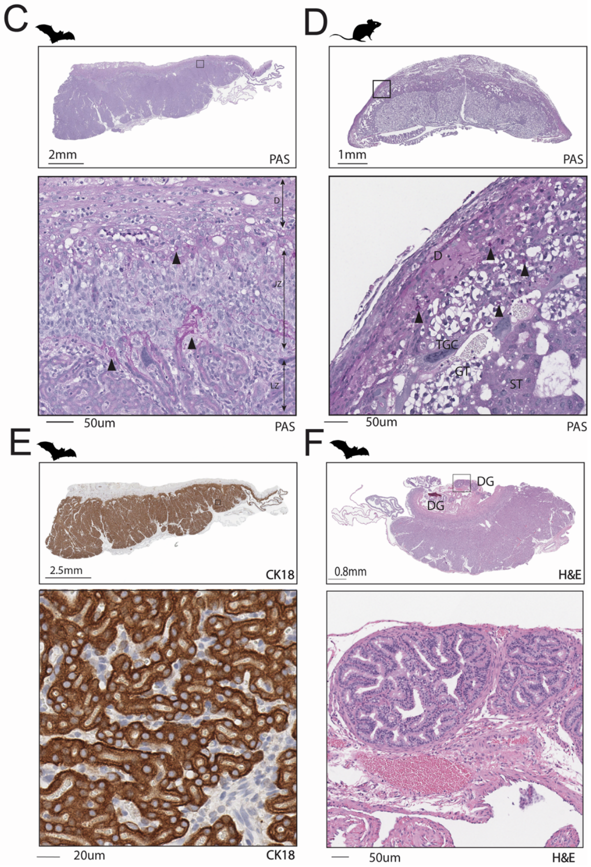

Figure S1. **Jamaican Fruit Bat Placental Sampling, Gestational Staging, and Histological Assessment, Related to Figure 1. (A)** Representative photographs of Jamaican fruit bat (Jfb) fetuses at the time of placental isolation, showing a range of developmental stages. **(B)** Table summarizing crown-rump length measurements, morphological features, estimated gestational ages corresponding to each fetus, based on established Jfb developmental staging criteria and fetal sex. **(C)** Periodic acid–Schiff (PAS) staining of Jamaican fruit bat (Jfb) placenta highlighting glycogen-rich trophoblast regions. **(D)** PAS staining of mouse placenta for comparison, showing typical labyrinth and spongiotrophoblast structures. D (decidua), TGC (trophoblast giant cell), GT (glycogen trophoblast), ST (spongiotrophoblast). **(E)** Immunohistochemistry (IHC) for KRT18 (CK18) in the Jfb placenta section marking trophoblast and epithelial cell populations. **(F)** Hematoxylin and eosin (H&E) staining of the bat decidua showing glandular structures (DG, decidual gland) embedded within the maternal decidua.

Figure S2

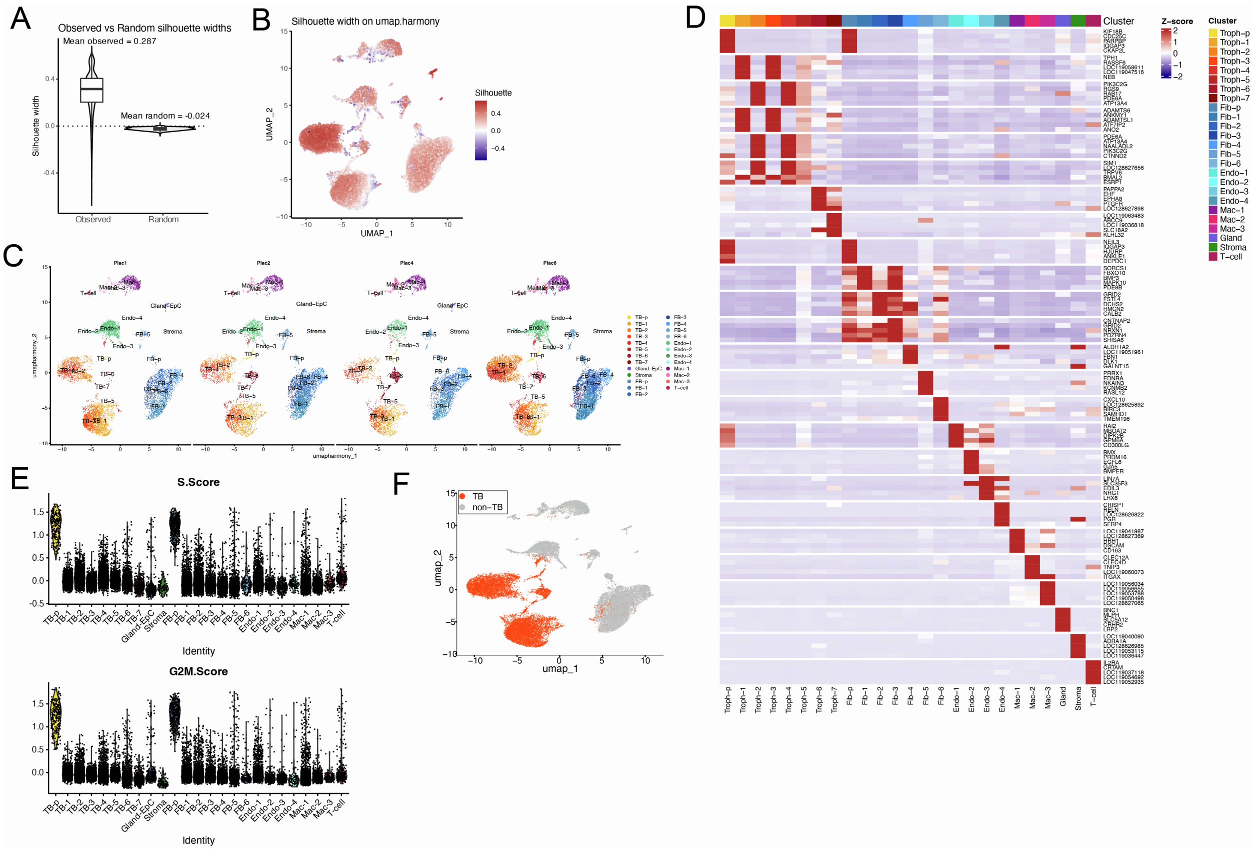

Figure S2. Defining Cellular Identities of the Jamaican Fruit Bat snRNA-seq dataset, Related to Figure 1. (A) Silhouette analysis comparing observed versus randomized cluster assignments reveals a positive shift in mean silhouette width (0.267 vs 0.024), indicating biologically meaningful structure. Boxes denote the interquartile range (IQR; 25-75th percentile), the horizontal line represents the median, and the whiskers show the 1.5x IQR. **(B)** UMAP plot overlaid with per-nucleus silhouette width highlights strong separation of major clusters following Harmony integration. **(C)** UMAPs showing cluster identities across unique samples overlaid with annotations based on marker expression. Clusters are named according to dominant lineage identity (e.g., Trophoblast, Endothelial, Fibroblast, Macrophage). **(D)** Heatmap of the top five uniquely enriched genes per cluster based on differential expression analysis (Seurat, logFC > 0.25, min.pct = 0.25). Z-scored expression highlights cell type–specific marker genes used for lineage assignments. **(E)** Cell cycle scoring across all clusters. Violin plots display S-phase and G2/M-phase scores, identifying proliferative populations, particularly within progenitor trophoblast and proliferating fibroblast clusters. **(F)** UMAP plot distinguishing trophoblast (TB, orange) versus non-trophoblast (non-TB, grey) clusters, based on marker gene enrichment and lineage annotation.

Figure S3

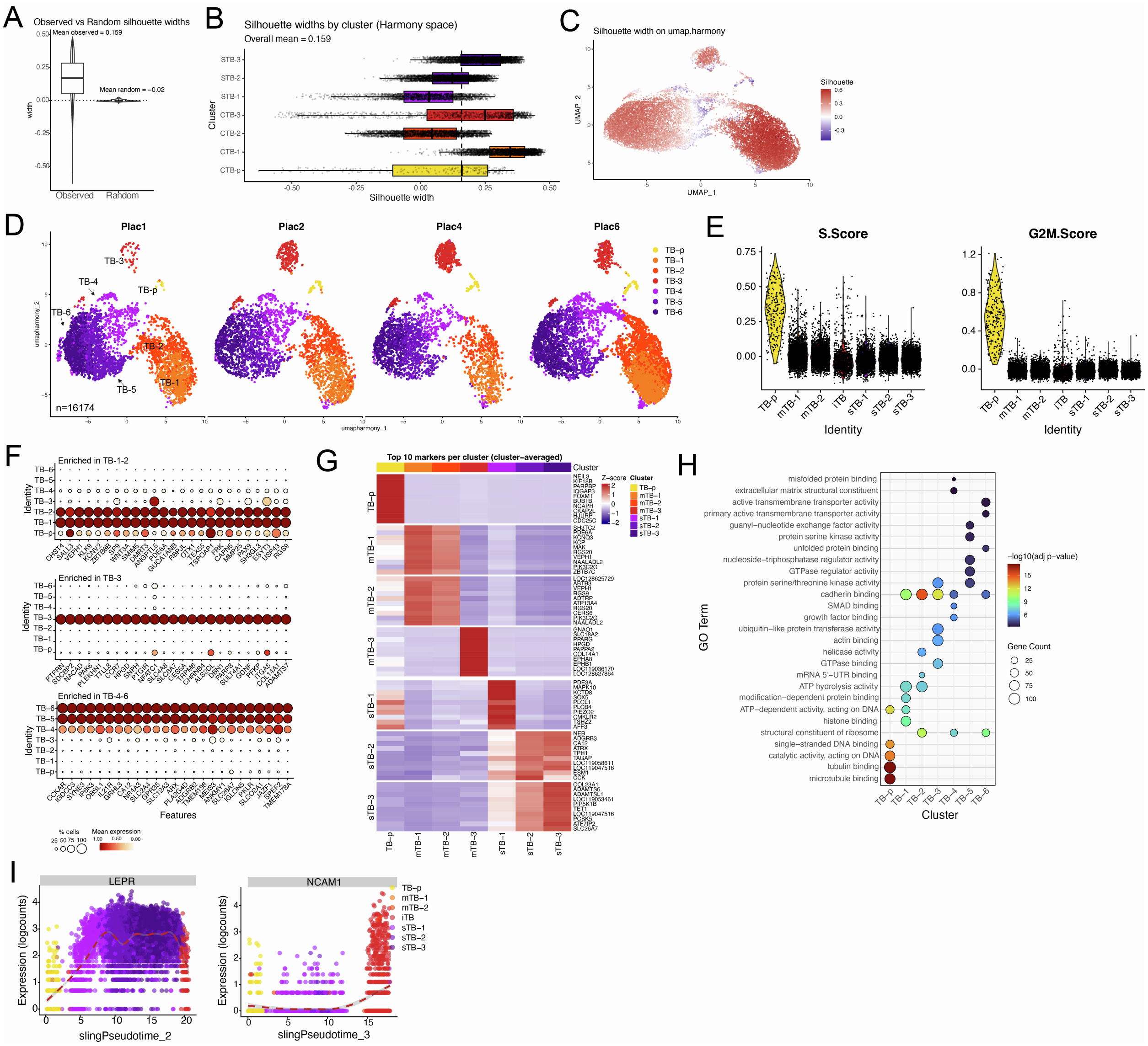

Figure S3. **Defining Cellular Identities of Trophoblast Populations in Jamaican Fruit Bat snRNA-seq dataset, Related to Figure 3. (A)** Violin plot comparing silhouette widths of observed clusters versus randomized cluster labels, showing strong biological structure (mean observed = 0.267; random = 0.024). Boxes denote the interquartile range (IQR; 25-75th percentile), the horizontal line represents the median, and the whiskers show the 1.5x IQR. **(B)** Silhouette widths per cluster in Harmony-corrected PCA space (overall mean ≈ 0.159), showing variability in internal cohesion across clusters. Boxes denote the interquartile range (IQR; 25-75th percentile), the horizontal line represents the median, and the whiskers show the 1.5x IQR. **(C)** UMAP colored by per-cell silhouette score computed in UMAP space, highlighting cohesive vs. diffuse clusters. **(D)** UMAPs of four placental samples showing cluster-specific distributions of trophoblast subpopulations. **(E)** Violin plots of S-phase and G2/M-phase cell cycle scores reveal proliferative status of trophoblast clusters. **(F)** Dot plots showing top cluster-enriched genes across all

trophoblast subtypes, identifying shared and unique transcriptional signatures. **(G)** Heatmap of top 10 differentially expressed genes per cluster (cluster-ordered), illustrating distinct marker gene expression across trophoblast subpopulations. **(H)** Gene Ontology enrichment (biological process and molecular function) for cluster-specific DEGs, highlighting functional pathways associated with each trophoblast state. **(I)** Slingshot trajectory analysis performed on PCA embeddings, using the proliferative TB-p cluster as the root, reveals pseudotemporal gene expression dynamics for key markers (e.g., *LEPR*, *NCAM1*) across trophoblast differentiation.

Figure S4

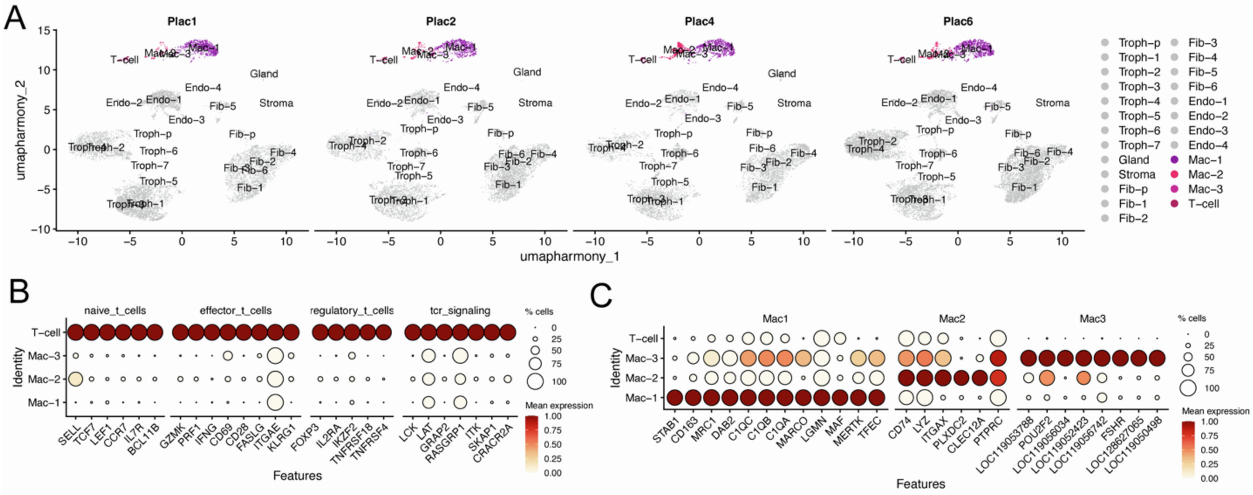

Figure S4. Defining Cellular Identities of Immune Cell Populations in Jamaican Fruit Bat snRNA-seq dataset, Related to Figure 4. (A) UMAP split by original sample identity (orig.ident) showing near-equivalent distribution of nuclei across all four biological replicates, confirming lack of major batch effects. Only immune cell subsets are colored. **(B, C)** Expression of canonical markers of T-cells (B) or macrophages (C) across clusters, showing heterogeneity within myeloid and lymphoid compartments.

Figure S5

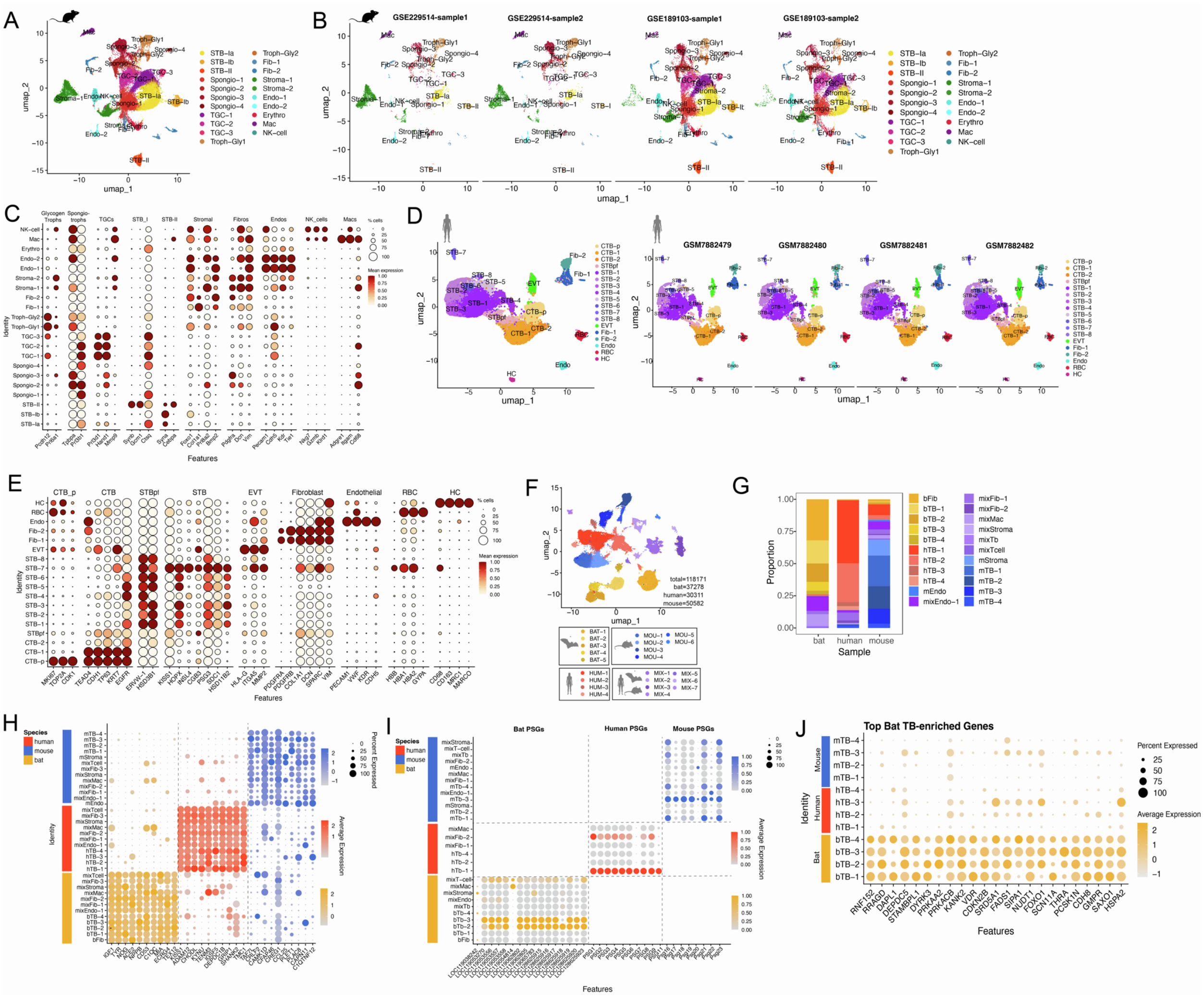

Figure S5. Cross-Species Integration and Cell Type Validation of Bat, Human, and Mouse Placental Datasets, Related to Figure 5. (A–B) UMAP visualization of mouse placenta nuclei, shown as a total dataset (A) and split by individual samples (B), validating the presence of major placental cell types based on clustering patterns. **(C)** Dot plot displaying canonical marker expression across identified mouse placental cell populations. **(D)** UMAP visualization of human placenta nuclei, shown as a total dataset (left) and split by individual samples (right), confirming major placental cell populations. **(E)** Dot plot of canonical marker expression across human placental clusters. **(F)** UMAP visualization of 22 integrated clusters across bat, human, and mouse placenta datasets. **(G)** Proportional contribution of each species to individual clusters, highlighting species-specific and mixed-species groups. **(H)** Dot plot showing the expression of the top species-enriched genes in bat (yellow-orange), human (red), and mouse (blue). (I) Dot plot

showing the expression of the species-specific PSGs in bat (yellow-orange), human (red), and mouse (blue) clusters.

Figure S6

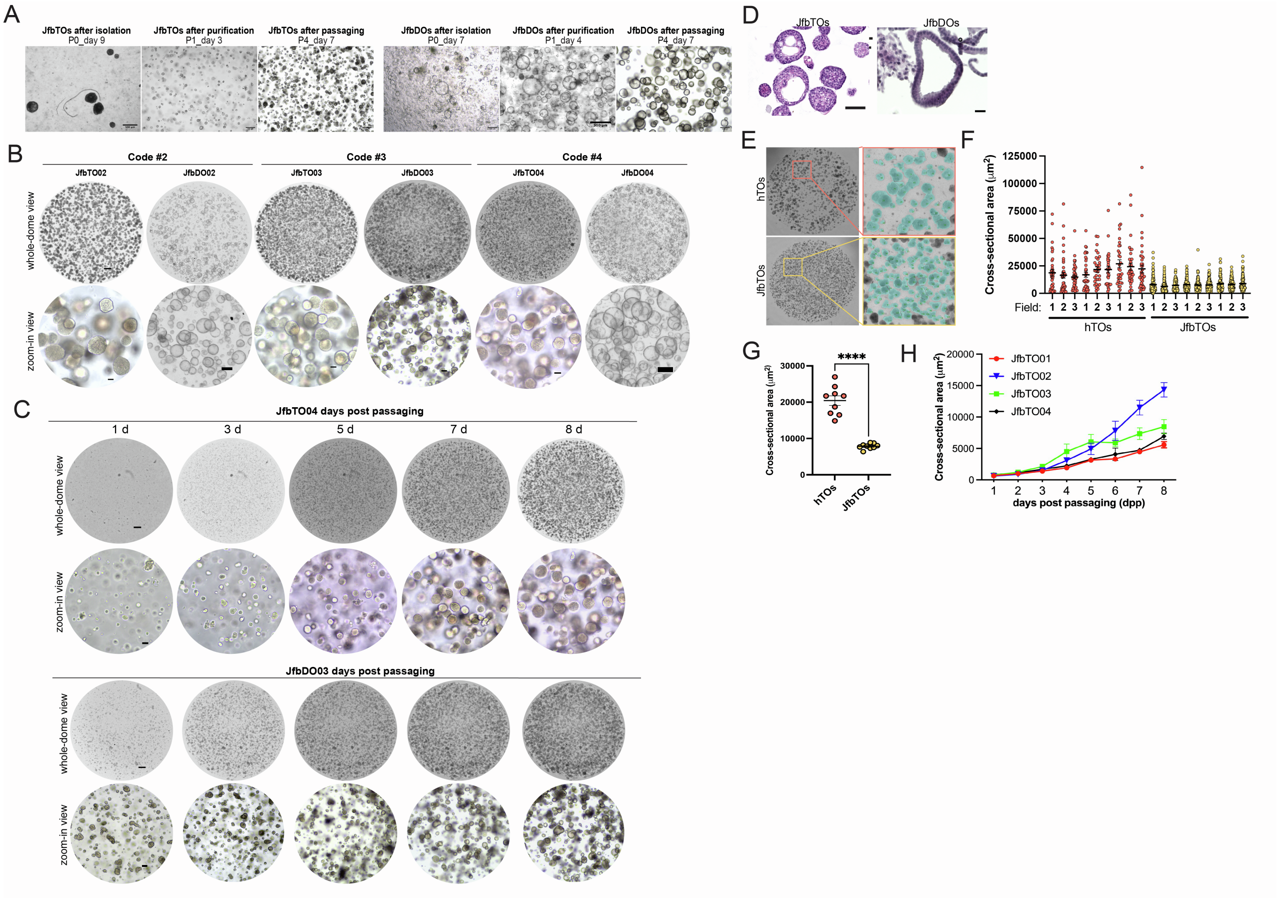

Figure S6: Imaging and Growth Metrics of Jfb Trophoblast and Decidual gland Organoids, Related to Figure 6. (A) Representative brightfield images of Jfb TOs (left panels) or DOs (right panels) at the indicated passage number (P) and days post-passaging (day_). Scale bar shown in images. **(B)** Representative brightfield images of three established Jfb TOs or DOs derived from 3 unique placental samples Top row is whole-dome images with scale bar, 500 μm, and bottom are zoomed to show organoid morphology with scale bar, 100 μm for TOs and 200 μm for DOs. **(C)** Representative brightfield images of Jfb TOs (top panels) or DOs (bottom panels) at the indicated days post-passaging. Top row is whole-dome images and bottom are zoomed to show organoid morphology. **(D)** Hematoxylin and eosin (H&E) staining of TOs and DOs, showing dense 3D architecture with internal cavities in TOs and a hollow cystic morphology in DOs. Scale bars, 100 μm**. (E)** Left panel, representative whole-dome brightfield images of hTOs (top) and JfbTOs (bottom); Right panel, zoomed representative 500 x 500 pixels squares for hTOs (top) and JfbTOs (bottom) cross-sectional area analysis, cyan colored organoid units are the ones chose for analysis based on optimized thresholding. **(F, G)** Quantification of organoid cross-sectional area across multiple fields of view (F) and between JfbTOs and hTOs (G). Data points represent the average cross-sectional area per field of view (three fields per Matrigel dome). ****$p < 0.0001$, two-tailed unpaired t-test with Welch's correction. **(H)** Growth curve of 4 independent JfbTO lines based on organoid cross-sectional area analysis at indicated timepoints.

Figure S7

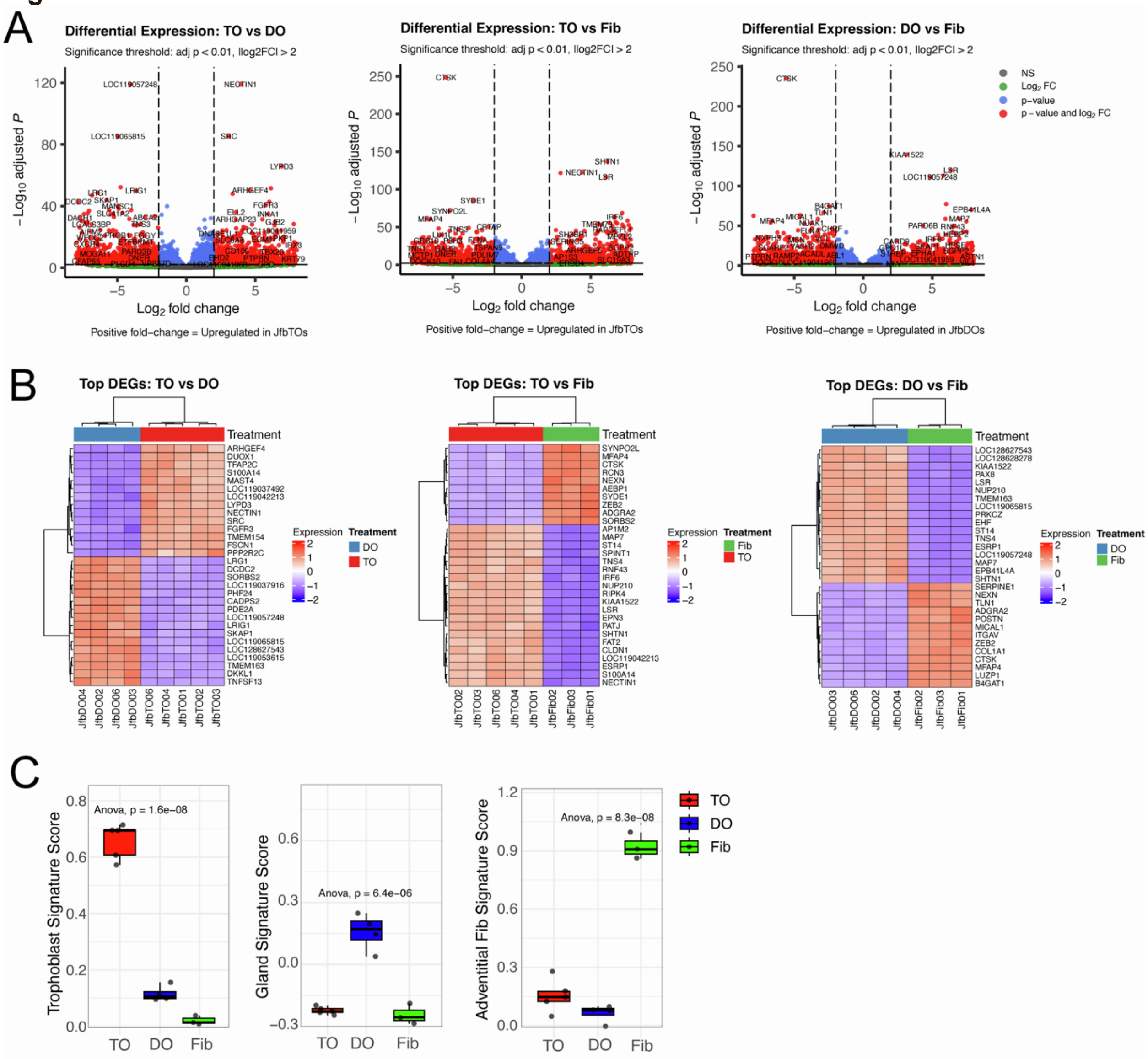

Figure S7. Bulk RNA-Seq Analysis of Jfb Trophoblast Organoids (TOs), Decidual Organoids (DOs), and Primary Fibroblasts, Related to Figure 6. (A) Volcano Plots of differentially expressed transcripts in TOs or DOs (left), TOs or fibroblasts (middle), DOs or fibroblasts (right) determined by DeSeq2 analysis. Blue circles represent transcripts that were not significantly changed and red circles represent transcripts significantly enriched, significance was set at p<0.01 and $\log_2$fold-change >± 2. **(B)** Heatmaps (based on $\log_2$ RPKM values) of top differentially expressed transcripts in TOs vs DOs (left), TOs vs fibroblasts (middle), DOs vs fibroblasts (right). Key at right and red indicates high level of expression, purple indicates low levels of expression. Hierarchical clustering is on top. (**C**) BoxPlots of trophoblast (left), glandular epithelial (middle), or adventitial fibroblasts (right) in TOs (red), DOs (blue), or primary placental fibroblasts (green). Boxplots representant the median (center line), interquartile range (box), data range within 1.5x the interquartile range. Each point denotes an individual sample, n=5 biological replicates of TOs, n=4 biological replicates of DOs, and n=3 biological replicates of TOs.

Figure S8

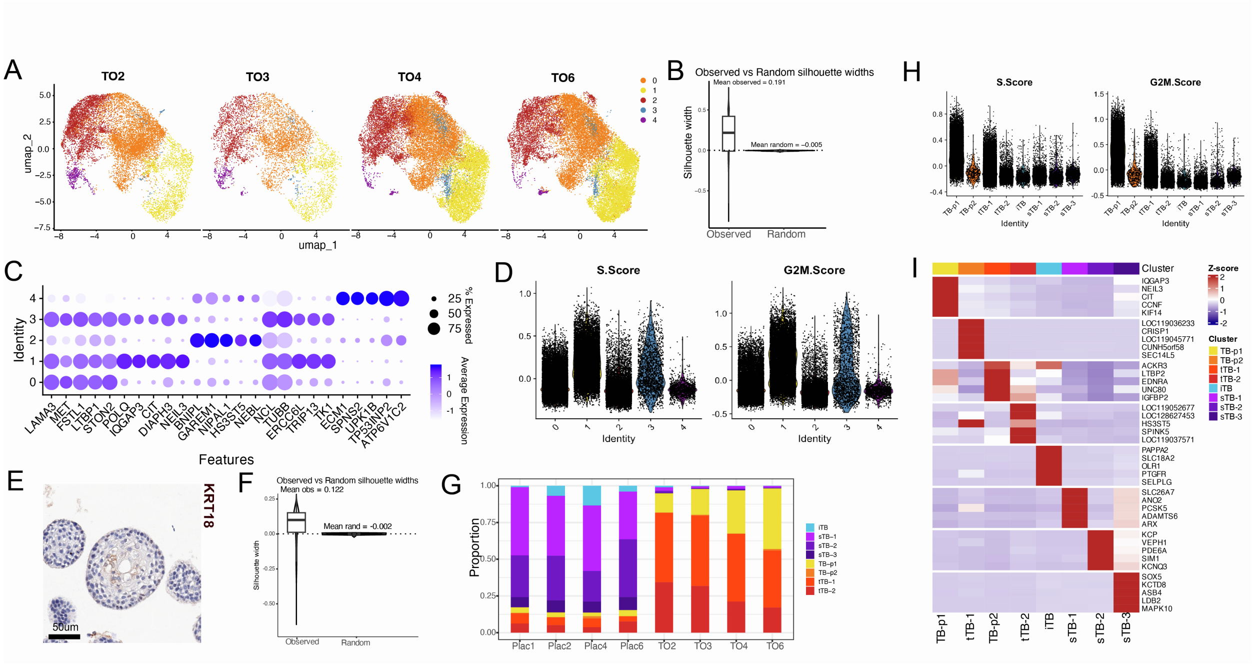

Figure S8. Defining Cellular Identities of JfbTO snRNA-seq dataset, Related to Figure 6. (A) UMAP visualization of snRNA-seq data from four independent TO cultures (TO2, TO3, TO4, TO6), revealing five transcriptionally distinct clusters. **(B)** Silhouette score analysis in TOs confirms meaningful clustering structure in TOs, with observed scores exceeding those of randomized labels. Boxes denote the interquartile range (IQR; 25-75th percentile), the horizontal line represents the median, and the whiskers show the 1.5x IQR. **(C)** DotPlot showing expression of top 5 enriched genes across TO-derived clusters. **(D)** Cell cycle scoring for S and G2/M phases reveals proliferative subsets, notably enriched in cluster 0 and 1 in TOs. **(E)** Representative immunohistochemistry for KRT18 protein expression in bat TOs validates distinct trophoblast cell populations. **(F)** Silhouette score analysis in integrated TO and tissue-derived trophoblasts confirms clustering structure, with observed scores exceeding those of randomized labels. Boxes denote the interquartile range (IQR; 25-75th percentile), the horizontal line represents the median, and the whiskers show the 1.5x IQR. **(G)** Stacked bar plot showing the relative proportions of each cluster across placental tissue and TO samples, demonstrating both shared and TO-enriched populations. **(H)** Cell cycle scoring for S and G2/M phases reveals proliferative subsets, notably enriched TO-enriched clusters **(I)** Heatmap of the top 5 cluster-specific marker genes, highlighting distinct molecular programs across TO-derived clusters.

Figure S9

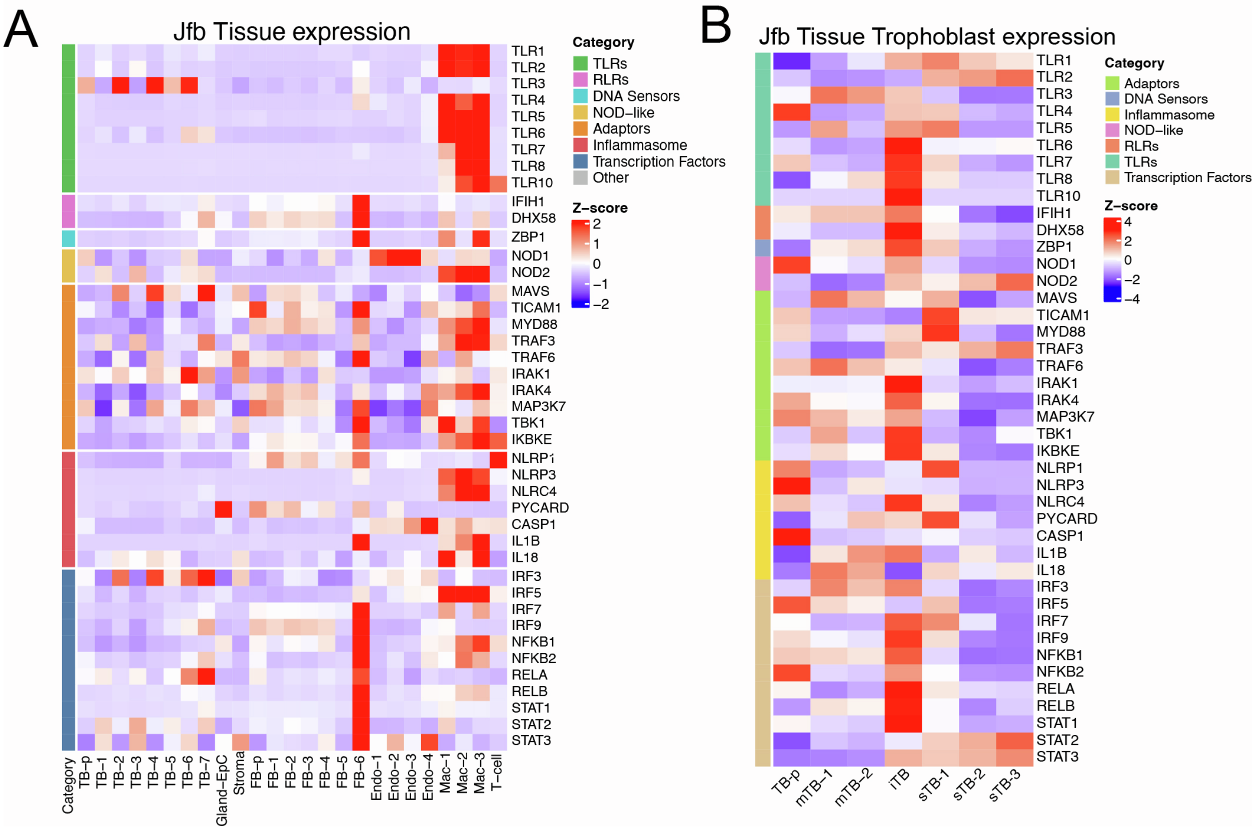

Figure S9. PRR expression in Jfb placental tissue and tissue-derived trophoblasts, Related to Figure 7. (A, B) Heatmap showing scaled average expression (Z-score) of key innate immune signaling genes including Toll-like receptors (TLRs), RIG-I–like receptors (RLRs), NOD-like receptors (NLRs), adaptor proteins, inflammasome components, and downstream transcription factors as well as markers of adventitial fibroblasts (in K) across distinct cell populations in the Jfb placenta (A) or tissue-derived trophoblasts (B). Genes are grouped by functional category, with color-coded annotations at left. Expression values were derived from SCT-normalized single-nucleus RNA-seq data and averaged per cluster.
